# Research Experiences via Integrating Simulations and Experiments (REVISE): A Model Collaborative Research Project for Undergraduate Students in CO_2_ Sorbent Design

**DOI:** 10.1021/acs.jchemed.3c01153

**Published:** 2024-02-22

**Authors:** Anthony Griffin, Neziah Smith, Mark Robertson, Bianca Nunez, Jacob McCraw, Haoyuan Chen, Zhe Qiang

**Affiliations:** † School of Polymer Science and Engineering, The University of Southern Mississippi, 118 College Drive, #5050, Hattiesburg, Mississippi 39406, United States; ‡ Department of Science, Copiah-Lincoln Community College Natchez Campus, 11 Co-Lin Circle, Natchez, Mississippi 39120, United States; § Department of Chemistry and Department of Physics and Astronomy, The University of Texas Rio Grande Valley, 1201 W. University Drive, Edinburg, Texas 78539, United States; ∥ School of Science and Engineering, Jones County Junior College, 900 S. Court Street, Ellisville, Mississippi 39437, United States

**Keywords:** Upper-Division Undergraduate, Graduate Education/Research, Interdisciplinary/Multidisciplinary, Collaborative/Cooperative Learning, Undergraduate Research

## Abstract

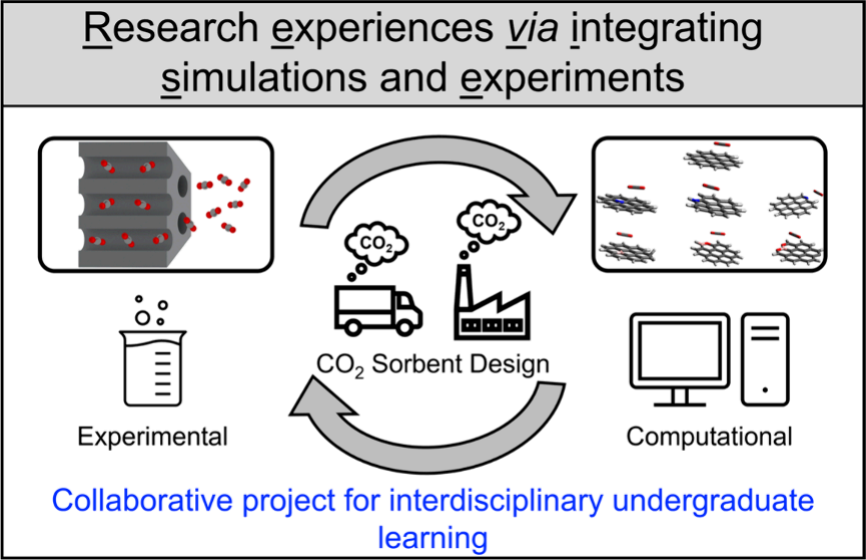

Undergraduate research experiences are an instrumental component of student development, increasing conceptual understanding, promoting inquiry-based learning, and guiding potential career aspirations. Moving one step further, as research continues to become more interdisciplinary, there exists potential to accelerate student growth by granting additional perspectives through collaborative research. This study demonstrates the utilization of a model collaborative research project, specifically investigating the development of sorbent technologies for efficient CO_2_ capture, which is an important research area for improving environmental sustainability. A model CO_2_ sorbent system of heteroatom-doped porous carbon is utilized to enable students to gain knowledge of adsorption processes, through combined experimental and computational investigations and learnings. A particular emphasis is placed on creating interdisciplinary learning experiences, exemplified by using density functional theory (DFT) to understand molecular interactions between doped carbon surfaces and CO_2_ molecules as well as explain underlying physical mechanisms that govern experimental results. The experimental observations about CO_2_ sorption performance of doped ordered mesoporous carbons (OMCs) can be correlated with simulation results, which can explain how the presence of heteroatom functional groups impact the ability of porous carbon to selectively adsorb CO_2_ molecules. Through an inquiry-focused approach, students were observed to couple interdisciplinary results to construct holistic explanations, while developing skills in independent research and scientific communications. This collaborative research project allows students to obtain a deeper understanding of sustainability challenges, cultivate confidence in independent research, prepare for future career paths, and most importantly, be exposed to strategies employing interdisciplinary research approaches to address scientific challenges.

## Introduction

A considerable emphasis in the development of undergraduate curricula toward engaging students through inquiry-based modules can be exemplified by the *National Research Council’s* education standards where inquiry is championed as the “heart of science and science learning”.^1^ Undergraduate research experiences can increase student knowledge, inform students about potential career possibilities, and promote more positive outlooks within their field of study.^2−4^ As students are able to take ownership of their research as well as experience the role of a scientist, an increase in educational value is observed when compared to conventional, verification-based studies.^5,6^ While there are many types of undergraduate research, most typically involve students working closely under a graduate/postdoctoral/faculty mentor with relatively defined roles.^7,8^ Interestingly, cross-subject collaboration between different groups, a common practice in the research community for promoting scientific discovery and knowledge, occurs less often between undergraduate researchers. By exposing undergraduates to collaborative research, students can become better cognizant for future career paths and obtain added learning.

Over the last few decades, the value of computational modeling has become increasingly recognized as these tools have been used to understand and predict behaviors of real systems and confirm phenomena observed through experimental results and hypotheses.^9^ Through this complementary relationship, experimental and computational collaboration has been demonstrated to accelerate the discovery and validation of fundamental mechanisms.^10^ With recent advances in the availability and reduced costs of student/community-friendly computational resources, integrating computational modeling research into undergraduate coursework and laboratories becomes increasingly popular.^11,12^ By leveraging computational power to explain chemical phenomena from experimental observations, students can obtain a further elevated fundamental understanding of their experimental work/results, while potentially developing complementary techniques and research skills.^13^ Collectively, there is an apparent and strong need to develop current undergraduate students through improving research experiences via integrating simulations and experiments (*REVISE*).

As sustainable environment development is and will continue to be a key research and education focus for future generations, design of collaborative research projects focused on the environment-material nexus can be a part of undergraduate training programs to help students identify challenges as well as obtain skills for problem solving.^14−17^ To this end, sorbent design for CO_2_ capture and understanding how material chemical composition impacts CO_2_ sorption performance is a highly relevant research project for undergraduate students in chemistry, materials, and chemical engineering programs/majors. Here, we present a collaborative undergraduate student-led research project that couples hands-on laboratory experiments with computational modeling for understanding design principles of CO_2_ sorbent nanomaterials on their capture performance. This project can be finished within an 8-week time frame with a 15–20 h per week lab workload commitment and is suitable for summer REU (research experience for undergraduate) students. Specifically, a team of undergraduate students from two different research groups, respectively, lead experimental and computational efforts, where the first group learns how to prepare and characterize doped ordered mesoporous carbon (OMC) materials while the second develops simulation models of synthesized products for explaining experimental observations. Through virtual meetings and regular updates, the team of undergraduates is exposed to computational and experimental research integration, which facilitates a comprehensive understanding. The learning objectives of this project primarily involve developing a conceptual understanding of sorbent design as well as obtaining experimental and computational skills, in addition to fostering the ability to couple interdisciplinary research approaches to construct solutions for prominent scientific challenges. The scientific objective of this collaborative research project is to understand the influence of heteroatom dopants and content levels on CO_2_ binding affinity for informed sorbent material design.

Furthermore, while this particular example was carried out by REU students, similar research projects with this “*REVISE*” concept can be employed in the broader chemical education community, such as upper division undergraduate laboratory courses with the potential to tune to different research foci. Specifically, the fundamental concepts in this project (material synthesis, adsorption behavior, and environmental sustainability concepts), coupled with the opportunity to develop research skills that are within the range of complexity for undergraduate courses and can be accomplished in a single lab session, have the potential to provide significant value in undergraduate training. Overall, this work provides an excellent opportunity to inspire and engage the next generation of STEM (science, technology, engineering, and math) researchers through focusing on an important technological and societal challenge, while introducing computational modeling and hands-on basic lab skills and sorption-related knowledge, effectively connecting general, organic, and physical chemistry.

## Learning Objectives

Overall, the scientific purpose of this laboratory experience is to investigate the CO_2_ sorption performance of doped OMC sorbents with varying heteroatom type and content level, while providing combined laboratory-computational research experiences to undergraduate students that act as a launch pad for them to discover research interests in computational modeling, materials synthesis, and fundamental physical chemistry. Specifically, the objectives include determining the impact of several heteroatom dopants (boron, nitrogen, phosphorus, sulfur) and heteroatom loading levels on the amount of CO_2_ adsorption of OMCs, in addition to coupling experimental results with computational modeling to develop a holistic understanding of dopant–CO_2_ interactions. This project was accomplished through collaborations between experimental and computational research teams with two cohorts throughout two years (details are provided in the Supporting Information), where the second cohort implemented improvements recommended from the first cohort. Specifically, two undergraduate students led experimental efforts and one undergraduate student led computational efforts in the first cohort. In the second cohort, three undergraduate students led experimental efforts and one undergraduate student led computational efforts. We note that undergraduate students, consisting of two community colleges, led the majority of research efforts with guidance from a graduate student mentor who mainly assisted with instrumentation for material characterization. The focus of incorporating students from community college backgrounds to research projects has been shown to be a key factor for “fostering the next generation” of scientists and engineers to make “chemical engineering broadly accessible” as well as promote a diversity of student trajectories.^18^

The main student learning objectives are listed below:

1. Obtain an understanding of the importance of environmental sustainability as well as overarching material (sorbent) design concepts for CO_2_ capture.
2. Obtain synthetic and characterization technique proficiency for porous materials (for the experimental team).
3. Understand and master the computational workflow that includes molecular model construction, geometry optimization, and binding energy calculation (for the computational team).
4. Obtain the ability to couple knowledge gained from laboratory and computational results to properly evaluate/explain a materials system to advance environmental sustainability efforts.

## Materials Preparation

CO_2_ sorbents were fabricated following an established protocol,^19^ where an ordered mesoporous polymer–silica composite is first prepared, including steps of cross-linking and calcination. Heteroatom dopants were then introduced through grinding prior to carbonization. The carbonized powders were then etched to remove silica and any byproducts, resulting in doped carbon sorbents. A full material preparation procedure is provided in the Supporting Information.

## Safety Hazards

While performing experiments, students must wear personal protective equipment at all times, including safety goggles, lab coats, gloves, and closed toed shoes. All reactions which evolve noxious and/or combustible chemicals must be performed in a fume hood. Special care must be taken when handling hydrochloric acid and potassium hydroxide due to their corrosiveness. Moreover, care should also be taken when handling tetraethyl orthosilicate (TEOS) as it is flammable and a skin and eye irritant. It is strongly recommended that all instructors and students go through each reagent’s MSDS (Material Safety Data Sheet) for more information prior to conducting experiments. During calcination and carbonization steps, the furnace and crucibles must be allowed to cool down to room temperature to avoid potential burn injuries. Moreover, the tube furnace must be equipped with an exhaust vent or placed in a fume hood to ensure exit gas, which contains byproducts (e.g., CO_2_) from the thermal degradation of compounds. When handling liquid nitrogen for setting up physisorption characterization, cryo-gloves, face protection, and a lab coat must be worn to reduce the risk and severity of cryogenic burns. Moreover, liquid nitrogen should only be handled in rooms with good ventilation.

## Implementation

This research project was first conducted during the summer/fall of 2022 with three undergraduate students leading experiment and computational efforts (nitrogen- and boron-doped samples). Specifically, students were given several weeks to develop an understanding of important concepts for sorbent material design as well as to learn necessary laboratory skills. This was then followed by them preparing and characterizing sorbent materials or developing molecular models of the sorbent devices binding efficiencies to CO_2_. Throughout this process, students maintained consistent collaboration through meetings to discuss findings as well as explain concepts to each other. Following this, students had the opportunity to explain fundamental design principles by coupling experimental and computational findings.

Points of improvement from the first cohort were found and addressed in the following summer of 2023 (focusing on phosphorus- and sulfur-doped samples), where this research project was further developed and more organized to an 8-week training program for four additional undergraduate students (specifically, REU projects). Notable changes in research project design from 2022 to 2023 include having a more inquiry-based approach by introducing additional heteroatom identities that allow for students to further hypothesize their varying impact on sorbent performance, promoting student-led discussion during weekly meetings focused on furthering conceptual understanding and challenging hypotheses, and encouraging additional literature review throughout the entire project timeline rather than just the first few weeks.

A 5E instructional model (engage, explore, explain, elaborate, and evaluate) was utilized to guide the design of this research project to promote inquiry-based learning.^20^ Through this evidence-based training approach, students were navigated through fundamental concepts and encouraged to explore and relate scientific concepts to their own experimental results in order to improve their understanding. Furthermore, as their conceptual understanding increased, they were further challenged to relate computational and experimental findings to assess material systems. The implementation of this research project is further discussed in more detail in the following sections as well as in the Supporting Information.

### Engagement

Within this section, students were introduced to important concepts of sorbent design principles for CO_2_ capture. This was achieved through literature review and collaborative discussions throughout 2 weeks. Background reading materials are provided in the Supporting Information. Important concepts discussed/introduced are highlighted below.

#### CO_2_ Capture and Remediation

Perpetually increasing atmospheric carbon dioxide (CO_2_) levels, stemming from the combustion of fossil fuels in various sectors, such as transportation,^21^ manufacturing,^22^ and electricity generation,^23^ have predominantly driven various distressing environmental developments, including increased food supply disruptions, extreme weather events, global warming, wildfires, and air pollution.^24,25^ To address grand-scale CO_2_ emission and curtail impending environmental and health challenges, the Paris Agreement was reached by 194 nations in 2015: a carbon neutral society (i.e., net-zero CO_2_ emission) must be achieved by 2050.^26^ A vital component toward this goal is developing CO_2_ capture technology which could also act as a catalytic cornerstone for further renewable energy conversion and electrochemical energy storage.^27,28^ While liquid amine-based CO_2_ sorbents (e.g., 2-aminoethanol (MEA), piperazine, pyrrolizidine-based diamines) have been demonstrated and adopted commercially, several challenges still exist in these systems,^29^ including significant energy costs for sorbent regeneration, corrosion, sorbent stability, and the production of harmful byproducts.^30^ Alternatively, solid sorbents for CO_2_ capture could require low energy penalties for regeneration in addition to the potentials of high stability, improved sorption capacity, and CO_2_ selectivity.^29,31^

#### Ordered Mesoporous Carbons (OMCs)

OMCs contain pores between 2 and 50 nm, which are promising CO_2_ sorbent materials due to their advantageous features of relatively high surface areas, highly accessible, uniform pore channels, and controllable matrix chemistry.^32^ In general, OMCs can be prepared through a soft-templating approach,^33^ which relies on the use of amphiphilic surfactant/copolymer templates to direct the nanostructure of carbon precursors (e.g., resol) through self-assembly, followed by cross-linking and carbonization. During carbonization, the templating agent can be thermally decomposed resulting in the formation of pores, while the precursor is converted to the final carbon framework. Another common OMC synthesis method is through hard-templating, which involves more steps than soft-templating and is described by several review papers.^34,35^ While these conventional strategies require several processing steps and expensive precursors that have as of yet limited their commercial usage, preparation of OMCs with commodity precursors and simplified manufacturing pathways have recently been developed to improve their economic competitiveness and potentially allow for their commercial application.^36,37^ As porous carbon is already used as a commercial sorbent material, the implementation of OMCs to field experiments and commercial applications has significant promise.

Moreover, incorporation of heteroatoms, such as nitrogen and sulfur, into the OMC framework can result in improved CO_2_ capture performance.^38^ For example, introducing nitrogen heteroatoms, with a pair of lone electrons, alters Lewis basicity of the carbon surface.^39^ These polar nitrogen sites can form strong pole–pole interactions with the quadrupole moment of CO_2_ molecules, which can improve material CO_2_ sorption capabilities. Utilization of heteroatom doping for controlling OMC physicochemical properties allows for the tailorable fabrication of sorbent materials toward desired performances. Many routes have been demonstrated for doping of OMCs with control over heteroatom content and type, such as the use of ammonia gas or other dopant materials during pyrolysis.^40,41^ These methods for OMC synthesis would allow one to create a controlled doped OMC-based CO_2_ sorbent design space to enable the investigation of how heteroatom functionality affects CO_2_ affinity to carbon surfaces.

### Exploration

**Figure 1:**
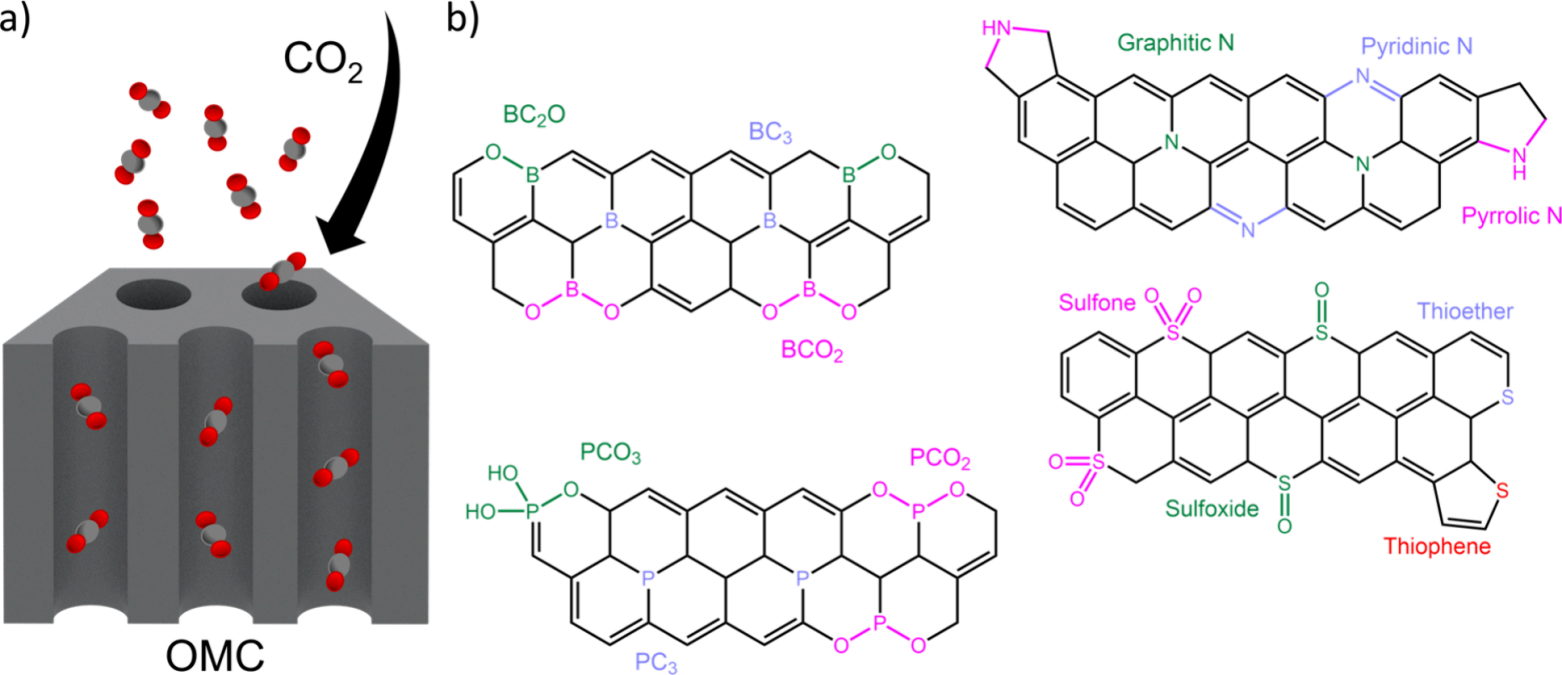
(a) Schematic illustration depicting the capture of CO_2_ by ordered mesoporous carbon sorbents and (b) predominant bond types of different heteroatoms within the carbon matrix.

Porous carbons with several heteroatom identities were prepared by undergraduate students throughout a three-week period following procedures outlined in the Supporting Information. Briefly, doped porous carbons can serve as CO_2_ sorbents,^42,43^ where pore surfaces serve as CO_2_ sorption sites and heteroatom doping can alter interactions between the carbon surface and sorbates (Figure 1a). The experimental portion of this research project includes the synthesis and characterization of doped OMC through a soft-templating approach following an established protocol^41^ in which the heteroatom doping type and content can be controlled through altering processing conditions (Figure 1b). Specifically, phenol resin, TEOS, and an amphiphilic surfactant (e.g., F127) are self-assembled into cylindrical nanostructures and subsequently cross-linked to prepare a resol-silica carbon precursor. The obtained material is then calcinated at 350 °C where the sacrificial organic template (F127) degrades, resulting in mesoporous carbon-silica. Details about OMC preparations through templating approaches, including self-assembly mechanisms, can be found in several review articles,^33−35^ which students are encouraged to read. Students can dope these nanostructured carbon precursors through pyrolysis of calcinated samples mixed with solid dopants, such as melamine for nitrogen doping, boric anhydride for boron doping, ammonium dihydrogen phosphate for phosphorus doping, and dibenzyl sulfide for sulfur doping. Students are encouraged to examine how different heteroatoms can be incorporated into carbon structures and hypothesize how each would impact the binding of CO_2_. The heteroatom identity and loading level are parameters that can be varied to establish a material design space for sorbent preparation. When the carbon precursor and solid dopants are thermally carbonized (∼800 °C) under inert gas, a synchronous synthesis of OMC and heteroatom doping occurs, which results in heteroatoms preferentially incorporating into the mesoporous carbon matrix. The silica particles in the carbon framework, derived from TEOS precursors, can reinforce the framework during simultaneous doping and carbonization (to prevent nanostructure collapse) and are subsequently etched in aqueous KOH solution which results in a high surface area, doped OMC.

The synthetic components of the research project were performed entirely by undergraduate students under the supervision of a graduate student mentor. All undergraduate students involved in the synthesis of this research project had very limited experience in organic chemistry laboratories or research. Following a week of important safety considerations and laboratory skills being demonstrated by a graduate student, undergraduate students were capable of performing all the tasks required in this research project. The experimental tasks in this research project allowed for the development of basic laboratory skills in addition to the introduction of fundamental concepts such as self-assembly and combinatorial material design. To investigate how heteroatom functionality and doping level affects CO_2_ capture, we focused on nitrogen heteroatoms here with controlled mass ratios of 0.5:1, 1:1, and 2:1 of dopant (melamine) to carbon precursor (calcinated products) as well as 2:1 boron, 1:1 sulfur, and 0.25:1 phosphorus-doped analogs. The experimental design of this research project was determined first by what was already available in the laboratory, but from those metal dopants, specific dopant identities/loading levels were discussed and determined by undergraduate students and advisors during the first meeting. While the dopant identities/loading levels described in this model research project provide room for undergraduate students to independently determine various chemical phenomena, there is significant flexibility depending on the interests of undergraduate students and the availability of laboratory inventories.

Following completion of doped OMC synthesis, undergraduate students can employ characterization techniques to understand material morphologies and pore textures, including physisorption interpreted by Brunauer–Emmett–Teller (BET) and nonlocal density functional theory (NLDFT) theory (Figures S1–S5). All physisorption characterization techniques were performed by undergraduate students under the supervision of a graduate student mentor. To confirm accuracy, undergraduate students would rerun the undoped sample following training to ensure consistent measurements were obtained. A commonly accepted method of characterizing surface area is through BET analysis, based on sample adsorption isotherm of a nonreactive species (e.g., nitrogen at 77 K). This method examines a range of pressures that spans the monolayer coverage of molecules for determining monolayer loading and subsequently specific surface area (see the Supporting Information, Background and Introductory Materials: Characterization). The nitrogen isotherm can be further utilized to determine pore size distribution through NLDFT theory, which interprets the adsorption isotherm in ideal pore geometries by employing classical fluid density functional theory. Here, we observe relatively high surface areas for all doped samples, while undoped OMC has a surface area of 1,449 m^2^/g (Table 1). Specifically, we found that the majority of doped samples had similar surface areas (∼1,200 m^2^/g), slightly reduced compared to the control (undoped) sample. However, two exceptions were observed, with N-OMC-7 and S-OMC-3 exhibiting slightly higher surface areas than the undoped control, which may be attributed to activation of the carbon framework with low levels of dopant present during the pyrolysis step. The heteroatom doping content for each sample was investigated through energy-dispersive X-ray spectroscopy (EDX) conducted on a scanning electron microscope (SEM), where a summary of loading levels is shown in Table 1. Here, nomenclature is used for OMC samples, which is x-OMC-y, in which x represents doped heteroatom type (N for nitrogen, B for boron, P for phosphorus, and S for sulfur) and y is the wt % of heteroatom content.

**Table 1:** (a) Surface Area, (b) Pore Size, and (c) Doping Content for Doped OMCs and an Undoped Control

Sample	BET Surface Area (m^2^/g)	Pore Size (nm)	Doping Content (wt %)
Control	1,449	6.5	
N-OMC-7	1,612	8.6	7
N-OMC-9	1,176	8.3	9
N-OMC-11	1,164	8.3	11
B-OMC-10	1,207	6.7	10
P-OMC-6	1,264	9.06	6
S-OMC-3	1,580	7.44	3

### Explanation

**Figure 2:**
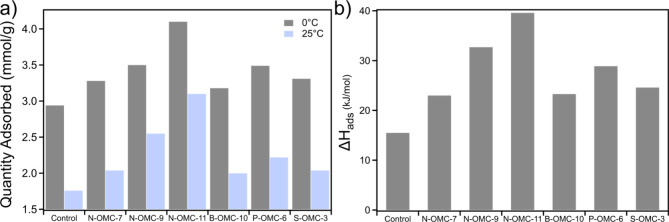
CO_2_ adsorption performance at (a) 0 and 25 °C and (b) gas selectivity calculated through Henry’s law, comparing CO_2_ and N_2_ adsorption capacities at room temperature under low pressure, for doped OMCs and an undoped control.

In addition to the preparation of sorbent materials, students also assessed the binding affinity of each species to determine sorbent design guidelines. In addition to this, students had guided discussions every week to not only discuss their own findings but also attempt to merge experimental and computational results to develop a better understanding of sorbent design. For example, to understand how doping of OMCs affect their sorbent–sorbate affinity for CO_2_ capture, CO_2_ sorption studies were performed at 0 and 25 °C. As shown in Figure 2a, it was found that increasing nitrogen content led to an increased sorption capacity of the system, with consistent increases at both measured temperatures. As nitrogen content increased, a steady increase in CO_2_ sorption capacity was observed. Furthermore, for the three additional heteroatom dopants, an increase in sorption capacity was also observed, which varied based on sorbent identity and loading level. Compared to the nitrogen-doped sample with similar heteroatom content (11 wt %), B-OMC-10 exhibited significantly lower CO_2_ sorption capacity, which only shows a slight increase over the control. The increased CO_2_ sorption performance for both nitrogen- and boron-doped samples can be attributed to enhanced Lewis acid/base interactions which has been reported in the literature,^39,44^ while different mechanisms may be involved. Additionally, the phosphorus-doped sample, with a loading of 6 wt %, had a similar CO_2_ sorption capacity to N-OMC-9, indicating a significant increase in sorption performance at a lower doping level. Finally, sulfur-doped carbon exhibited a CO_2_ sorption improvement similar to N-OMC-7 even with a lower loading content of 3 wt %.

The sorption performance of the nitrogen-doped samples was enhanced by greater pole–pole interactions as well as stronger Lewis basicity in the carbon imparting enhanced interactions with CO_2_. In comparison, for boron-doped OMCs, the electron-accepting boron heteroatoms can directly interact with CO_2_, acting as an active site for CO_2_ capture. However, their CO_2_ sorption capacity improvement is significantly less pronounced than the nitrogen-doped counterparts. In this education-focused research project, these hypotheses were developed by participating undergraduate students, which can be directly tested using simulation approaches and will be discussed in the following section. Furthermore, selectivity of OMC-based sorbents toward CO_2_ over N_2_ can be determined through calculating the Henry’s Law constant, including the initial slope (<0.2 bar) of adsorption, for each gas molecule. The ratio of the slopes represents the CO_2_/N_2_ selectivity, which is a key material property for industrial applications. Particularly, a high CO_2_/N_2_ selectivity value is important for addressing CO_2_ capture from postcombustion flue-gas from power plants, as these gas streams contain approximately 80–90% N_2_ and 10–20% CO_2_. In Figure 2b, we observe an anticipated trend of increased selectivity as nitrogen doping increases from 15:1 for the control to 23:1, 32:1, and 39:1 for 7, 9, and 11 wt %, respectively. In comparison, the boron-doped sample (26:1) also exhibited an increase compared to the control, albeit lower than nitrogen-doped samples with comparable loading. With CO_2_ being adsorbed ∼39 times the amount of nitrogen at room temperature for the optimal sorbent system, the enhanced interactions between the doped carbon surface and CO_2_ guest molecules, due to the presence of heteroatom doping, is further confirmed. Moreover, phosphorus and sulfur doping also result in improvements in selectivity, consistent with their impact on CO_2_ sorption where they behave similarly to N-OMC-9 and N-OMC-7, respectively.^45,46^ Through these exercises, students became familiarized with selectivity calculations as well as developed an understanding of how to extract valuable information from isotherm data.

### Elaboration

**Figure 3:**
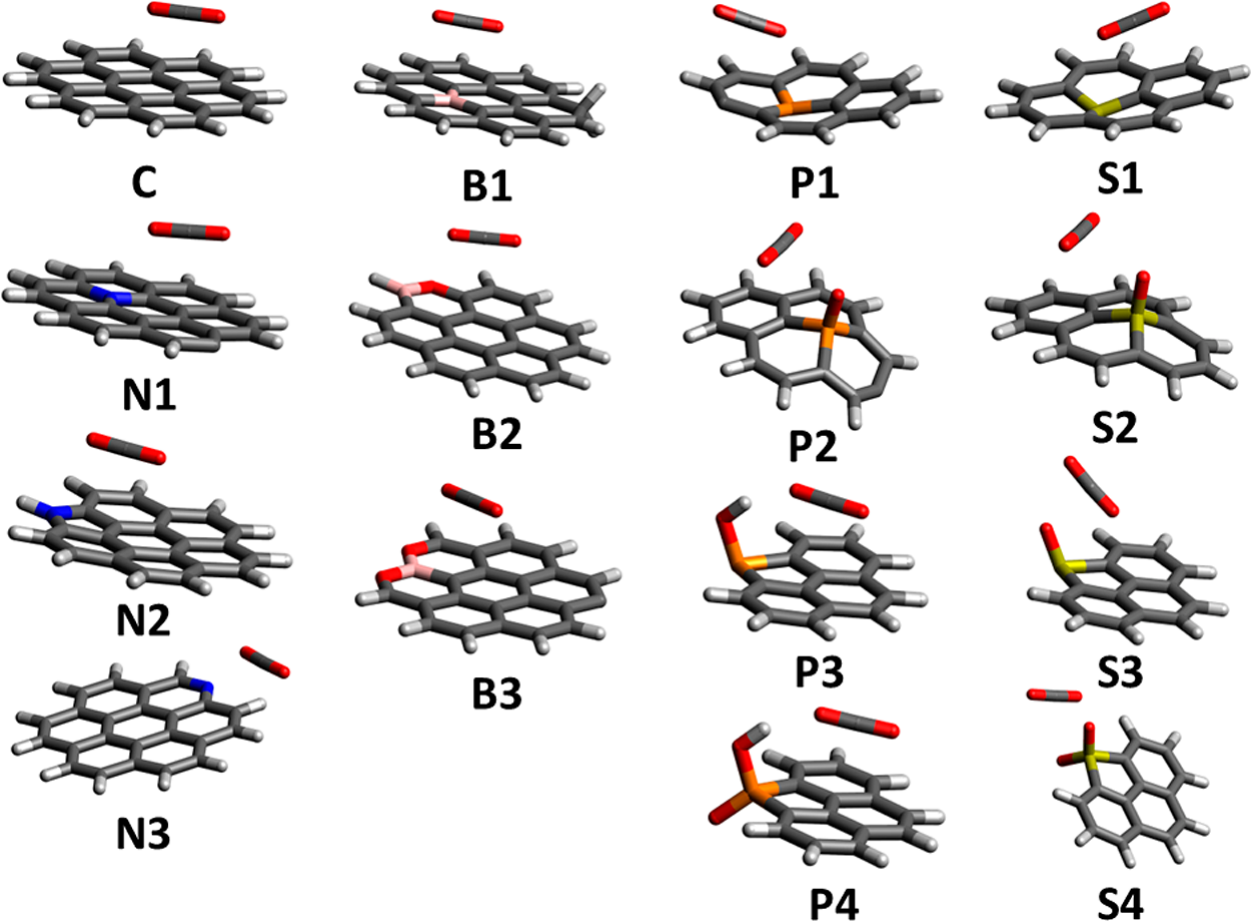
DFT-calculated most favorable CO_2_ binding mode in all molecules. Color code for elements: H, white; B, pink; C, gray; N, blue; O, red; P, orange; S, yellow.

To build upon experimental results and better understand fundamental sorbent design principles, computational modeling was carried out to provide an atomistic level mechanistic picture of CO_2_ adsorption in the OMCs. Molecular models as shown in Figure 3 were constructed based on the structural motifs shown in Figure 1b. The sulfur-doped calculations are from our previous work.^47^ As shown in Table 2, nitrogen doping leads to much stronger CO_2_ binding compared to carbon, while boron doping only has a moderate effect. Also, phosphorus and sulfur doping have similar enhancements on CO_2_ binding energy. This is in excellent agreement with the experimental results discussed above. We note that each of these density functional theory (DFT) calculations usually takes only minutes to a few hours on supercomputers (the Lonestar6 supercomputer under Texas Advanced Computing Center is used in this study), which is suitable for undergraduate students performing summer research as many supercomputers in the U.S. can be accessed by college students free of charge after a short research proposal is approved. Students can use free software, such as Avogadro and WebMO Basic (or commercial software such as GaussView and WebMO Pro, if available), to construct the molecular models, generate the input file for Gaussian, and visualize/analyze the output file after the calculation is finished. The communication between students’ computers and the supercomputer can be done using secure shell protocol (SSH) and secure copy protocol (SCP) tools such as PuTTY and WinSCP. If the students use MacOS or Linux, the login and file transfer can be done in the default terminal included in the operating system. Several reports outlining the major steps with detailed protocols have been included in the Supporting Information.^48−50^ Compared to traditional chemistry experiments in the lab, these computational “experiments” offer great flexibility in time and location. This can clearly demonstrate to undergraduate students the power of modern computational chemistry in solving real-world problems.

**Table 2:** CO_2_ Binding Energy (in kcal/mol) in the Most Favorable Binding Mode for All Molecular Structures Calculated from DFT

Molecule	Energy	Molecule	Energy	Molecule	Energy	Molecule	Energy
C	–3.34	B1	–3.91	P1	–4.31	S1	–3.53
N1	–3.99	B2	–3.37	P2	–5.54	S2	–4.38
N2	–4.08	B3	–3.28	P3	–5.33	S3	–5.39
N3	–5.28			P4	–5.48	S4	–4.59

From the DFT results, the structure with the strongest CO_2_ binding in nitrogen-doped OMCs is pyridinic nitrogen (N3). As shown in Figure 3, N3 is the only structure in which CO_2_ prefers to bind to the heteroatom on the “*side*”, rather than staying “*flat*” on top of the surface. Note that, for each molecule, we investigated several different binding poses as initial structures, and the ones shown in Table 2 and Figure 3 are the most energetically favorable binding mode for each molecule. “*Side*” binding modes were also tried for several other molecules, but they either have weaker binding than “*flat*” or went to “*flat*” during geometry optimization. It is only favorable in N3 because the nitrogen atom in N3 is only bonded to two atoms, leaving an open space for CO_2_ binding. This binding is likely driven by electrostatics, as the negative partial charge on nitrogen can attract the positive partial charge on carbon (in CO_2_). These electrostatic-driven “*side*” bindings are often stronger than the van der Waals-driven “*flat*” bindings, as shown in our previous work on sulfur-doped carbon.^47^ On boron-doped OMCs, CO_2_ binding is weaker than on nitrogen-doped OMCs and always takes the “*flat*” binding mode, as none of the boron-containing structure motifs creates an “open” boron atom. For phosphorus- and sulfur-doped OMCs, the strongest binding structures have similar CO_2_ binding energies and are close to N3 (Table 2), which agree with experimental findings. Interestingly, the strong electrostatic “*side*” binding in S3 and S4, which was observed in previous work,^47^ changed in P3 and P4, as replacing an S(IV)/S(VI) atom with P(III)/P(V) will change a S/P=O to a S/P–OH, which enables hydrogen bonding with CO_2_. From our meetings, this analysis of computational results has helped students revisit and gain a better understanding of their general/organic/physical chemistry knowledge.

### Evaluation

This research project can be performed by a collaborative team of undergraduate or REU students in 8 weeks. The majority of the synthesis and characterization steps do not require extensive time commitments from students and can be flexible depending on laboratory time constraints. The computational part is even more flexible, as it can be done on the students’ own laptops once the remote connection to the supercomputer is set up. We found students can understand basic physical sorption concepts and OMC synthesis concepts through reading introductory materials (provided in the Supporting Information). For lab work, students were supervised by graduate student mentors, who need to be present to ensure safety protocols are followed in addition to explaining the experimental setup. Furthermore, the introduction of computational studies and implementation of computational modeling are designed to provide a suitably challenging exercise to guide initial computational skill development (provided in the Supporting Information).

For assessing learning outcomes, students are requested to present their findings through a preliminary and final presentation (expectations and rubric are provided in the Supporting Information, Student Assessment), which can gauge their conceptual understanding of environmental sustainability, CO_2_ pollution, and important CO_2_ sorbent design parameters in order to achieve learning objective 1. Specifically, students are asked to describe the impact of CO_2_ on climate changes and provide a brief overview about the development of sorbent technology. To assess learning objective 2, students are gauged on their synthetic and characterization techniques first through the use of a standard sample following training. Following successful characterization, a second assessment is carried out during the preliminary presentation where students are required to sufficiently discuss important concepts of each synthetic step and characterization technique, as well as present initial findings for each result to ensure proper proficiency for all laboratory skills. Lastly, during the final presentation, this proficiency is also assessed. The computational modeling of doped OMCs can be evaluated through examination of calculated binding energies and binding modes during the preliminary and final presentations to achieve learning objective 3. To assess learning outcome 4, the preliminary presentation was carried out to gauge conceptual understanding in addition to the ability to integrate experimental and computational results into an overarching material system evaluation. This was found to be the most difficult task for undergraduate students to proficiently understand conceptually, though based on feedback from this presentation, students were able to achieve this learning objective, which was assessed once more in the final presentation.

From the first cohort of students, we found from these assessments (see the Supporting Information) that this research project was well received, but we also found room for improvement. Specifically, the major challenge students faced was coupling results together, especially between experimental and computational groups. This had been anticipated as all participants had at most taken one organic chemistry lecture course and had limited research backgrounds. To assist with this, we implemented weekly meetings with all undergraduate trainees to attempt to establish a common background, where students summarized their findings of each week. While these exercises were shown to succeed in developing important concepts during the engagement portion of the project which was focused on literature review, we found that the first cohort had difficulty in explaining results and analyses during the middle of the project. Though results made logical sense to students within their respective teams, the diffusion of this knowledge between experimental/computational groups was fairly difficult. This was primarily discovered during the preliminary presentation, and to improve upon this and achieve learning outcome 4, a greater emphasis was placed during weekly meetings to ensure every undergraduate student understood one another’s updates. We found that students were hesitant and apprehensive of asking questions in fear of appearing unintelligent. By encouraging students to ask questions, dedicating half the allotted time for questions and discussions between groups, and challenging each student to ask a certain number of questions each meeting, we found this significantly improved the dissemination of data analyses and discussion as well as achieved the goal of “*REVISE*”. Additionally, we found students were interested in investigating additional heteroatom dopants/loading levels to observe potential interactions and sorbent effects. To further promote this inquiry-focused research project, in the second cohort, we added additional heteroatom dopants rather than primarily investigating a single heteroatom at varied loadings as concluded in the first cohort, though these were limited to phosphorus and sulfur in order to not overly convolute underlying mechanisms and allow students an adequate design space to further speculate for CO_2_ binding hypotheses. Further work may include coupling 2 or more dopants or allowing students to consider new heteroatoms that may further improve sorbent performance. From a modeling perspective, we also identify that students can simulate more sorbate gases beyond CO_2_, such as methane (CH_4_) and sulfur dioxide (SO_2_), which are also toxic and/or greenhouse gases. This would also allow students to further leverage the power of computational research to understand and predict rational materials design. Additional lessons we learned that can be used to improve further student (*REVISE*) learning experiences include giving students samples containing randomized heteroatom identities and loading levels, where students can make hypotheses about material composition and performance, and including additional heteroatoms as well as doping levels following completion of the initial experiment to further expand upon the sorbent design space if time permits.

Overall, from our learning assessments with students, we found this research project has been well received. Students from both teams have appreciated the integration of hands-on laboratory and computational learning environment, though a few potential points of revision have been identified to further shift to a more inquiry-based research experience. This experimental research training process was found by students to significantly improve their research/learning independence during their research experience and build confidence in their conceptual understanding as well as their laboratory and inquiry skills.

## Discussion

As undergraduate curricula continue to implement inquiry-based strategies to promote improved learning outcomes and diversity, undergraduate research has remarkable potential to not only develop the next generation of researchers but also become a cornerstone of chemical education as a whole. As undergraduates can take ownership of their research, students can become more engaged both in and out of the classroom with inquiry-based educational strategies. This opportunity is further strengthened as the ability of undergraduate students to conduct independent research has grown considerably in recent years, evidenced by numerous publications with undergraduate students being the primary authors. Moreover, academic research has continued to utilize cross-subject collaboration due to the value in combining expertise in various fields to advance new discoveries and attain holistic scientific understanding. Though this brings great promise in expanding the scope of undergraduate students to various fields, this practice of research collaboration and its role in enriching undergraduate student understanding has been explored only to a relatively limited extent. This work provides a prototypical example of academic collaboration, involving experimental results and computational modeling to drive comprehensive understanding of new phenomena. The model research project was focused on demonstrating how involvement in this collaborative space can stimulate the development of undergraduate students. The scientific topic of CO_2_ capture sorbent design is important and timely, considering the global efforts in improving environmental sustainability and decarbonization. While this project was through an 8-week REU program, this methodology of combining experiment and computational research in a collaborative undergraduate project, as well as developing an inclusive and diverse learning environment, can be easily expanded to various undergraduate student course/research modules, including REU students and senior/honors theses, as well as incorporated into upper division laboratory courses. For example, learning computational skills has now been introduced in many undergraduate courses, and therefore, instructors could have opportunities to design an inquiry-based project to allow students to practice experimental and simulation techniques to enable a comprehensive understanding of chemical data science exploration as well as the determination of chemical reaction kinetics and activation energy. Moreover, this “*REVISE*” strategy, while demonstrated with a CO_2_ sorbent design project, is envisioned to provide flexibility within the constraints of this research focus but can also be expanded toward additional research foci of faculty interested in embracing this collaborative research approach to undergraduate education.

## Conclusions

This work demonstrates a model collaborative undergraduate research project and concept that integrates laboratory experiments and computational modeling for providing interdisciplinary research and learning experiences for students, aimed at OMC sorbent design for CO_2_ capture. This collaborative research project provides undergraduate students the opportunity to connect interdisciplinary skills to propose holistic conclusions, while developing important conceptual understandings focused on environmental sustainability. We find this project enables students to become familiar with real-world challenges, be exposed to several fields of chemical research, and be guided to connect experimental and computational results to ascertain a comprehensive understanding of structure–property relationships.

## Supporting Information Available

Notes for instructors, lesson plan, outcome insights and future plans, background and introductory materials, recommended reading materials, student assessment, representative nitrogen adsorption, EDX, and CO_2_ adsorption results, and additional computational results (PDF; DOCX)
