# Supplementary material for: Research Experiences via Integrating Simulations and Experiments (REVISE): A Model Collaborative Research Project for Undergraduate Students in CO2 Sorbent Design
Source: J Chem Educ. 2024 Feb 22;101(3):1096–105. doi: 10.1021/acs.jchemed.3c01153 (PMC10938636; doi:10.1021/acs.jchemed.3c01153)
Supplement: Supplementary file 1 — ed3c01153_si_001.pdf [file ed3c01153_si_001.pdf]

Supporting Information for

Research experiences via integrating simulations and experiments (REVISE): A model undergraduate research project for CO_2 sorbent design

Anthony Griffin[1], Neziah Smith[2], Mark Robertson[1], Bianca Nunez[3], Jacob McCraw[4], Haoyuan Chen[3,*], Zhe Qiang[1,*]

[1] School of Polymer Science and Engineering, The University of Southern Mississippi, 118 College Drive, #5050, Hattiesburg, Mississippi 39406, USA

[2] Department of Science, Copiah-Lincoln Community College Natchez Campus, 11 Co-Lin Cir, Natchez, Mississippi 39120, USA

[3] Department of Chemistry, Department of Physics and Astronomy, The University of Texas Rio Grande Valley, 1201 W University Drive, Edinburg, Texas 78539, USA

[4] School of Science and Engineering, Jones County Junior College, 900 S Court St, Ellisville, MS 39437, USA

Corresponding Author

* Z. Q. Email: zhe.qiang@usm.edu

* H. C. Email: haoyuan.chen@utrgv.edu

Table of Contents

Notes for Instructors 2
Lesson Plan, Outcome Insights and Future Plans 3
Background and Introductory Materials 4
Recommended Reading Materials 11
Student Assessment 12
Representative Experimental and Computational Data 14

Notes for Instructors

Instructors should be aware of the general procedures for synthesis and doping of OMCs, as well as the characterization methods for physisorption and energy-dispersive X ray spectroscopy (EDX) which can be found in the Background and Introductory Materials section. It is recommended the instructor give recommended reading materials the week prior to an experiment, in addition to giving a brief introduction on experimental setup. This research project was designed to be successfully accomplished within an eight-week period with 15-20 hours of time committed per week, suitable for teams of undergraduate students including research experience for undergraduates (REU) students, but may also be extended to a project course for individual junior and senior undergraduates. Through this collaborative education-focused research project, student understanding can be facilitated by experimental and computational research, though this requires consistent meetings (which could be virtual if needed) as well as regular updates between collaborators. It is recommended that these occur at least once per week to ensure a comprehensive understanding if fostered between students. The following section shows demonstrates

outcome insights and future plans, showcasing the activities and learning outcomes expectations throughout this project as well as future plans.

Lesson Plan, Outcome Insights and Future Plans

Lesson	Activity	Expectation
1	Literature review of sorbent design for CO2 capture.	Learning Outcome 1
2	Synthesis and crosslinking of ordered mesoporous carbon (OMC) precursor.	Learning Outcome 2
3	Calcination, doping, and carbonization of OMC precursor	Learning Outcome 2
4	Construction of molecular models of pure and doped carbons on computers.	Learning Outcome 3
5	Characterization of doped OMCs (BET & SEM/EDX). Geometry optimization of CO_2 binding on the molecular models using DFT	Learning Outcome 3
6	Calculation of CO_2 binding energy for all the optimized structures using DFT	Learning Outcome 3
7	Analysis of experimental and computational results	Learning Outcome 4
8	Preparation and delivery of lab report and presentation	Learning Outcome 4

Through integrating experimental and computational research, undergraduate students are exposed to interconnected research approaches which guides student learning and fosters a comprehensive understanding. This collaborative research project is focused on exposure of students to these strategies, though future work could investigate integrating both activities into a future REU project where students could develop both skills.

In the first cohort of students, three students were tasked with investigating the effect of loading level of one heteroatom dopant (nitrogen) on sorbent performance as well as comparing it with a second dopant (boron). These material systems were chosen to simplify behaviors and allow for clear relationships to be devised primarily by undergraduate student discussion between experimental and

computation groups. Specifically, two undergraduate students were involved in the experimental group and one undergraduate student in the computation group. A room for improvement determined from this first cohort was that expanding the material system would allow for additional material behaviors that would further promote inquiry-driven learning. Based of this, the second cohort in the following summer probed several heteroatom dopants (adding phosphorus and sulfur) in addition to the initial experiments. This cohort had three undergraduate students in the experimental group and one undergraduate student in the computation group. This was found to convolute underlying mechanisms and require students to further spec ulate for hypotheses, though by coupling computational results to experimental findings as well as having weekly meetings between undergraduate students, these behaviors were clearly discussed and analyzed.

Background and Introductory Materials

CO_2 Capture

The rise of anthropogenic greenhouse gases released into the atmosphere is a worldwide crisis, due to not only the environment impact, including global warming, extreme weather patterns, and the threat of mass extinction, but also in its complexity as mitigation requires addressing the combustion of fossil fuels, which are used in several business sectors, such as manufacturing, transportation, and electricity generation. Specifically, carbon dioxide (CO_2) emissions need to be diminished to address these impending challenges. The Paris Agreement in 2015, agreed upon by 194 nations, aimed at setting a goal to limit global warming at a maximum of 2 °C above preindustrial levels and pursue efforts to further limit it to 1.5 °C. This value was determined as a target that would potentially avoid the negative, irreversible impacts climate change would have on society. To achieve this, global CO_2 emission levels must be reduced by 45% by 2030 from levels in 2010 and a carbon neutral society must be achieved by 2050. Not only does this necessitate creative solutions to reduce the combustion of fossil fuels by industries and

human society, but it also requires creative solutions to further minimize emission levels. Strategies to capture CO_2 focus on post combustion methods, where CO_2 is collected from the emission source and gas is cooled down and passed through a treatment process that reduces impurity concentrations. These include solvent adsorption, adsorption with solid sorbents, and membrane separation. Of these, solid sorbents are desirable due to their advantages of fast kinetics, long term stability, low energy demand for regeneration, high adsorption and selectivity at ambient conditions, and ease of handling.

Nanomaterials as sorbents

Several solid sorbent technologies have been developed over the past few decades, including zeolites, porous organic polymers, covalent organic frameworks, metal-organic frameowrks, and porous carbons. These rely on adsorption, where CO_2 is attached to the surface of the sorbent. This physical process benefits from the use of nanomaterials due to their physical and chemical properties. Their nano-scale size confers unique characteristics, with fairly high surface reactivity, stability, and the ability to be functionalized to tailor affinity towards specific target molecules. Of these technologies, ordered mesoporouc carbon (OMC) is particularly interesting due to its accessible, uniform pore channels, relatively high surface areas, and tunable carbon matrix. Conventional OMC production relies on a soft-templating strategy, where a carbon precursor (resol) is blended with an amphiphilic surfactant template to direct development of nanostructure. Following self-assembly, the material is crosslinked and carbonized. At these elevated temperatures the crosslinked carbon precursor is converted to carbon while the templating agent is degraded, resulting in a ordered porous carbon matrix.

Tailoring the sorbent design for CO_2 capture can be achieved by functionalization of the OMC matrix with heteroatom doping. By doping with boron or nitrogen, the sorbent capture performance can be modulated, depending on the chemical interaction with the target molecule. Doping of nitrogen into a carbon matrix, for example, can introduce Lewis basicity and form strong interactions with CO_2

molecules. Heteroatom doping of OMCs have been achieved by introducing ammonia gas or solid metal oxides during pyrolysis, which allows for the development of a sorbent design space with heteroatom identity and loading levels being controllable variables to explore how functionality can impact sorbent performance.

Density functional theory

Chemistry is mostly about electrons. The electronic structure of a molecule determines many of its chemical properties, such as reactivity. Getting the electronic structure of a molecular system requires quantum mechanical calculations such as solving the Schrödinger equation, as electrons are microscopic particles that obey quantum mechanics. Density functional theory (DFT) is an efficient way to solve the Schrödinger equation. The basic idea is that the (ground-state) properties of a molecular system depend and only depend on its electron density (first Hohenberg-Kohn theorem). The correct electron density is the one that makes the system reach its lowest possible electronic energy (second Hohenberg-Kohn theorem). Based on these two theorems, we can obtain the correct electron density of any molecular system by minimizing its electronic energy. With the correct electron density, we can then calculate any chemical properties we want.

The problem here is that, how can we calculate the electronic energy from the electronic density? This requires a functional--a common mistake among students who are new to computational chemistry is to call DFT “density function theory” rather than “density functional theory”. A function takes numbers as input and gives numbers as output, while a functional takes functions as input and still gives numbers as output. Here, a density functional $E[\rho]$ takes the electron density as input, which is a 3-dimensional function $\rho = \rho(x,y,z)$ that gives the probability to find an electron at a certain position (x,y,z), and gives the electronic energy as output, which is a number E. So far, we do not have a universal density functional that always gives the exactly correct result, but many good density functionals have been developed and

can give reliable results for chemical systems. They rely on approximate theories and/or empirical parameter fitting. The density functional we used in this work, called M06-2X, is a widely used one that has excellent performance in predicting the structure and energy of organic molecules.

Safety considerations

Students must wear personal protective equipment at all times during experiments, including safety goggles, lab coats, gloves, and closed toed shoes. All reactions which evolve noxious and/or combustible chemicals must be performed in a fume hood. Special care must be taken when handling hydrochloric acid and potassium hydroxide due to their corrosiveness. Moreover, care should also be taken when handling tetraethyl orthosilicate (TEOS) as it is flammable as well as being a skin and eye irritant. It is strongly recommended that all instructors and students go through each reagent's MSDS (Material Safety Data Sheet) for more information prior to conducting experiments. During calcination and carbonization steps, the furnace and crucibles must be allowed to cool to avoid potential burn injuries. Moreover, the tube furnace must be equipped with an exhaust vent or placed in a fume hood to ensure exit gas, which contains byproducts (e.g. CO_2) from thermal degradation of compounds. When handling liquid nitrogen for setting up physisorption characterization, cryo-gloves, face protection, and a lab coat must be worn to reduce the risk and severity of cryogenic burns. Moreover, liquid nitrogen should only be handled in rooms with good ventilation.

Materials

Phenol (>99%), sodium hydroxide (NaOH), hydrochloric acid (HCl; ACS reagent, 37%), potassium hydroxide (KOH), tetraethyl orthosilicate (TEOS; >98%), poly(propylene oxide)-*block*-poly(ethylene oxide)-*block*-poly(propylene oxide) triblock copolymer (Pluronic F127; PEO_{106}–PPO_{70}–PEO_{106}), boric anhydride (98%), ammonium dihydrogen phosphate (98%), dibenzyl sulfide (95%), and melamine (99%) were

obtained from Sigma-Aldrich and employed as dopants. Ethanol (190 proof) was obtained from Decon Labs, formaldehyde (37 wt% in H_2O; contains 10−15% methanol as a stabilizer) was purchased from TCI, and deionized (DI) water was obtained with a Milli-Q IQ 7003 ultrapure lab water purification system.

OMC fabrication

To a 50 mL round bottom flask, dissolve 2.4 g of Pluronic F127 in a mixture of 12 g of ethanol and 1.5 g of 0.2 M HCl. Heat the solution to 42°C and stir for 1 h until a homogenous mixture is obtained. Following this, add 7.5 g of resol solution (20 wt% in ethanol) and 3.12 g of TEOS and stir at 42 °C for 2 h. Cast the prepared solution onto petri dishes and dry overnight at room temperature. Crosslink the film at 100 °C for 24 h and calcinate at 350 °C for 2 h under a N_2 atmosphere using an MTI Corporation OTF-1200× tube furnace. After calcination, mix the calcinated sample with the desired dopant at the desired mass ratio by physically grinding with a mortar and pestle. Then, carbonize these mixtures at a rate of 1 °C/min to 600 °C followed by 5 °C/min to 800 °C under N_2 atmosphere. Etch the carbonized powders with a 2 M KOH solution and refresh daily for 3 days to remove silica and byproducts. Following this, wash several times with deionized water, centrifuge, and dry overnight at 105 °C.

Characterization

Gas physisorption behavior was determined here with a Micromeritics Tristar II 3020, though it can be adapted to alternative equipment. For CO_2 sorption, the experiment will be run at two temperatures, 25 °C and 0 °C. Room temperature physisorption requires no additional setup, while 0 °C requires the use of an ice bath.

For N_2 sorption, the experiment will be carried out at room temperature and 77 K by exposing samples to liquid nitrogen. The surface area of samples will be obtained from the 77K N_2 experiment through the Brunauer−Emmett−Teller (BET) equation:

$$\frac{p}{X[\left(\frac{p_o}{p}\right)-1]}=\frac{1}{X_mC}+\frac{C-1}{X_mC}\left(\frac{p}{p_o}\right) \quad \text{Equation 1}$$

where p and p_o is the equilibrium and saturation pressure of adsorbate, X_m is the monolayer capacity (or the volume of gas adsorbed at standard temperature and pressure), and C is the BET constant. To determine the specific surface area, the BET equation is plotted at relative pressures (p/p_o) between 0.2-0.3 which by BET theory should be a straight line. The monolayer capacity is found from the gradient and intercept, which is then used to determine the total surface area by using the cross-sectional area of the molecule with the following equation:

$$s_{total}=\frac{X_mN_s}{V} \quad \text{Equation 2}$$

where V is the molar volume of the adsorbed gas and N_s is Avogadro's number, or 6.02×10^{23} molecules/mol. The specific surface area is then calculated from the total surface area over the mass of sample used. This theory relies on several assumptions, such as having a homogenous surface, infinite adsorption at saturation, and limited molecular interactions. Based off the representative experimental results that are consistent with previous reports, increasing doping content is observed to lead to a reduced surface area of OMC materials. The slight increase of surface area at the lowest nitrogen level can be attributed to NH_3-induced carbon activation, which is a gaseous byproduct upon melamine decomposition. The control and all doped samples exhibited type IV nitrogen adsorption isotherms, which are consistent with mesopore formation, as well as displayed uniform, monomodal pore size distributions, indicating the nanostructures of all OMC samples are ordered. Following this, the pore size distribution will be determined through non-local density functional theory (NLDFT) theory, which interprets the adsorption isotherm in ideal pore geometries by employing classical fluid density functional theory. Using an appropriate model for describing cylindrical pores and carbon surface in our system, the resulting pore size distribution can be obtained. For simplicity, instrument analysis software with a cylindrical model is used for fitting of the model to experimental data as it is not a trivial mathematical process. In the representative experimental results, the averaged pore sizes of all the nitrogen-doped samples are slightly

higher than the control, whereas the boron-doped sample is nearly identical to the control. This may be explained by nitrogen doping (which nitrogen atom has an increased atomic size than carbon) swelling the carbon matrix, resulting in slight pore expansion during carbonization. The selectivity of sorbents toward CO_2 over N_2 at room temperature can be investigated through the Henry's Law constant. This value can be calculated by placing the initial slope (<0.2 bar) of adsorption for CO_2 over N_2, which demonstrates the amount of carbon dioxide adsorbed compared to nitrogen for a sorbent system. Finally, elemental composition of samples can be probed through energy-dispersive X ray spectroscopy (EDX) was conducted on a Zeiss Ultra 60 field-emission scanning electron microscope (SEM). Based off the representative experimental results, samples with increasing dopant mass ratios exhibited a clear trend of increasing doping content, indicating the facile and robust nature of this synthetic approach for preparing doped OMC.

Assessing representative experimental results, it was found that increasing nitrogen content from 7 wt% to 9 wt% and 11 wt%, CO_2 sorption capacity at 1 bar and 25 °C improves from 2.0 to 2.6 and 3.1 mmol/g, respectively. At 0 °C, performance is further enhanced to 3.3, 3.5, and 4.1 mmol/g, respectively. The control sample, undoped OMC, exhibited a sorption capacity of 1.8 and 2.9 mmol/g at 25 °C and 0 °C, respectively. Notably, while the surface area of the control and lowest nitrogen level sample are higher, they do not exhibit a higher CO_2 sorption capacity than nitrogen-doped OMC samples containing greater nitrogen content. This result indicates that a strong influence of heteroatom functionality on CO_2 affinity and final sorbent performance which overrides the variations in surfaces areas.

Computational Methods

Atomistic molecular models for N- and B-doped OMCs were first constructed on computers with the coronene molecule (denoted as "Molecule C") as the starting point. Three N-doped molecules ("Molecule N1/N2/N3") and three B-doped molecules ("Molecule B1/B2/B3") were constructed to represent the structural motifs shown in Figure 1. N1/N2/N3 correspond to graphitic N/pyrrolic

N/pyridinic N, while B1/B2/B3 correspond to BC_3/BC_2O/BCO_2. When necessary, the amount of hydrogen atoms was adjusted (+1 or -1) to maintain the charge neutrality of the molecule. For P-doped OMCs, four structures ("Molecule P1/P2/P3/P4") were constructed by selecting four S-doped structures from previous works ("Molecule S1/S2/S3/S4") and replacing S/S=O with P/P-OH, followed by hydrogen adjustment when necessary. All structures were optimized using density functional theory (DFT) calculations with the M06-2X density functional and the def2-SVP basis set.

On each of the eleven optimized structures (C, N1/2/3, B1/2/3 and P1/2/3/4), one CO_2 molecule was added at different positions with different orientations. Each of these complexes was then optimized using the same density functional and basis set but also with the DFT-D3 dispersion correction to account for the van der Waals interaction between CO_2 and the adsorbent molecule. In some cases, different starting structures led to the same optimized structure. Finally, for each optimized complex, a counterpoise-corrected single-point energy calculation was performed using the same density functional and dispersion correction but with a larger basis set (def2-TZVP) to obtain the final CO_2 binding energy on each molecular model with higher accuracy. This computational protocol established in previous works showed good agreement with current experiments. All calculations were performed using Gaussian16 (revision A.03) on the Lonestar6 supercomputer at the Texas Advanced Computing Center.

Recommended Reading Materials

(1) Sholl, D. S.; Lively, R. P. Seven Chemical Separations to Change the World. *Nat.* **2016**, *532* (7600), 435–437.

(2) D'Amato, G.; Vitale, C.; Lanza, M.; Molino, A.; D'Amato, M. Climate Change, Air Pollution, and Allergic Respiratory Diseases: An Update. *Curr. Opin. Allergy Clin. Immunol.* **2016**, *16* (5), 434–440.

(3) The Paris Agreement. https://unfccc.int/process-and-meetings/%0Athe-paris-agreement/the-paris-agreement. (accessed 2023-01–10).

(4) Dunstan, M. T.; Donat, F.; Bork, A. H.; Grey, C. P.; Mü, C. R. CO2 Capture at Medium to High Temperature Using Solid Oxide-Based Sorbents: Fundamental Aspects, Mechanistic Insights, and Recent Advances. *Chemical Reviews*. **2021**. *121* (20), 12681-12745.

(5) Ryoo, R.; Joo, S. H.; Kruk, M.; Jaroniec, M. Ordered Mesoporous Carbons. *Adv. Mater.* **2001**, *13*

(9), 677–681.
(6) Qiang, Z.; Xia, Y.; Xia, X.; Vogt, B. D. Generalized Synthesis of a Family of Highly Heteroatom-Doped Ordered Mesoporous Carbons. *Chem. Mater.* **2017**, *29* (23), 10178–10186.
(7) Kelley, E. W. LAB Theory, HLAB Pedagogy, and Review of Laboratory Learning in Chemistry during the COVID-19 Pandemic. *J. Chem. Educ.* **2021**, *98* (8), 2496–2517.
(8) Sotomayor, F. J.; Cychosz, K. A.; Thommes, M. Characterization of Micro/Mesoporous Materials by Physisorption: Concepts and Case Studies. *Acc. Mater. Surf. Res* **2018**, *3* (2), 34–50.
(9) Robertson, M.; Obando, A. G.; Nunez, B.; Chen, H.; Qiang, Z. Upcycling Mask Waste to Carbon Capture Sorbents: A Combined Experimental and Computational Study. *ACS Appl. Eng. Mater.* **2022**, *1* (1), 165–174.
(10) Dos Santos, T. C.; Mancera, R. C.; Rocha, M. V. J.; Da Silva, A. F. M.; Furtado, I. O.; Barreto, J.; Stavale, F.; Archanjo, B. S.; De Carneiro, J. W. M.; Costa, L. T.; Ronconi, C. M. CO2 and H2 Adsorption on 3D Nitrogen-Doped Porous Graphene: Experimental and Theoretical Studies. *J. CO2 Util.* **2021**, *48*, 101517.
(11) Qiang, Z.; Guo, Y.; Liu, H.; Cheng, S. Z. D.; Cakmak, M.; Cavicchi, K. A.; Vogt, B. D. Large-Scale Roll-to-Roll Fabrication of Ordered Mesoporous Materials Using Resol-Assisted Cooperative Assembly. *ACS Appl. Mater. Interfaces* **2015**, *7*, 44.
(12) Leach, A. R. *Molecular Modelling: Principles and Applications*, 2nd ed.; Pearson, 2001.
(13) Weigend, F. Accurate Coulomb-Fitting Basis Sets for H to Rn. *Phys. Chem. Chem. Phys.* **2006**, *8* (9), 1057–1065.
(14) Zhao, Y.; Truhlar, D. G. The M06 Suite of Density Functionals for Main Group Thermochemistry, Thermochemical Kinetics, Noncovalent Interactions, Excited States, and Transition Elements: Two New Functionals and Systematic Testing of Four M06-Class Functionals and 12 Other Functionals. *Theor. Chem. Acc.* **2008**, *120*, 215–241.
(15) Weigend, F. Accurate Coulomb-Fitting Basis Sets for H to Rn. *Phys. Chem. Chem. Phys.* **2006**, *8* (9), 1057–1065.
(16) Grimme, S.; Ehrlich, S.; Goerigk, L. Effect of the Damping Function in Dispersion Corrected Density Functional Theory. *J. Comput. Chem.* **2011**, *32* (7), 1456–1465.
(17) Weigend, F.; Ahlrichs, R. Balanced Basis Sets of Split Valence, Triple Zeta Valence and Quadruple Zeta Valence Quality for H to Rn: Design and Assessment of Accuracy. *Phys. Chem. Chem. Phys.* **2005**, *7* (18), 3297–3305.
(18) Robertson, M.; Obando, A. G.; Nunez, B.; Chen, H.; Qiang, Z. Upcycling Mask Waste to Carbon Capture Sorbents: A Combined Experimental and Computational Study. *ACS Appl. Eng. Mater.* **2022**, *1* (1), 165–174.
(19) Frisch, M. J.; Trucks, G. W.; Schlegel, H. B.; Scuseria, G. E.; Robb, M. A.; Cheeseman, J. R.; Scalmani, G.; Barone, V.; Petersson, G. A.; Nakatsuji, H.; Li, X.; Caricato, M.; Marenich, A. V.; Bloino, J.; Janesko, B. G.; Gomperts, R.; Mennucci, B.; Hratchian, H. P.; Ortiz, J. V.; Izmaylov, A. F.; Sonnenberg, J. L.; Williams-Young, D.; Ding, F.; Lipparini, F.; Egidi, F.; Goings, J.; Peng, B.; Petrone, A.; Henderson, T.; Ranasinghe, D.; Zakrzewski, V. G.; Gao, J.; Rega, N.; Zheng, G.; Liang, W.; Hada, M.; Ehara, M.; Toyota, K.; Fukuda, R.; Hasegawa, J.; Ishida, M.; Nakajima, T.; Honda, Y.; Kitao, O.; Nakai, H.; Vreven, T.; Throssell, K.; Montgomery, J. A., Jr.; Peralta, J. E.; Ogliaro, F.; Bearpark, M. J.; Heyd, J. J.; Brothers, E. N.; Kudin, K. N.; Staroverov, V. N.; Keith, T. A.; Kobayashi, R.; Normand, J.; Raghavachari, K.; Rendell, A. P.; Burant, J. C.; Iyengar, S. S.; Tomasi, J.; Cossi, M.; Millam, J. M.; Klene, M.; Adamo, C.; Cammi, R.; Ochterski, J. W.; Martin, R. L.; Morokuma, K.; Farkas, O.; Foresman, J. B.; Fox, D. J. Gaussian 16 Rev. A.03. Gaussian, Inc., Wallingford, CT, **2016**.

Student Assessment

Student Expectations for Oral Presentation

To assess student's grasp of important concepts, ability to perform research tasks and acquire important results related to the research project, and ability to analyze and correlate experimental/computational results, students are asked to perform an oral presentation at the end of the research project in week 8. Important background topics include environmental sustainability, CO_2 pollution and sorbent devices, and sorbent design principles, students

At the end of 4th week, students are expected to present a preliminary presentation with a background section that should be fairly close to being finalized, an ongoing work section that should show the progress of results/analysis within the first few weeks, and a future work section that outlines the plan for the rest of the research project. Following this preliminary presentation, students are given feedback from mentors to improve on prior to the final presentation. Additionally, after edits are made based off feedback, students have a discussion with mentors to ensure the development of good fundamentals in a presentation including a well-constructed PowerPoint slide deck, ability to present, and ability to answer questions. A full rubric for the presentation is available below, which all attendees are expected to fill out and students are also expected to self-assess themselves.

The preliminary presentation should be 7.5 minutes long with a 5 minute Q & A period. The final presentation should be 12 minutes with a 5 minute Q & A period. The presentation structure should include:

- Background introducing important concepts and current challenges
- The problem statement and goal of the research
- Outline of experiments
- Results and Discussion
- Conclusions and Future Work
- Acknowledgements

Presentation Rubric

Criteria	Emerging (1 pt)	Developing (2 pt)	Proficient (3 pt)	Exemplary (4 pt)	Total
Organization and Structure	• Presentation is very difficult to follow and is unfocused<br>• Presentation is lacking a significant amount of required material<br>• No/poor transitions between slides/topics	• Presentation is slightly difficult to follow and rambles in certain points<br>• Presentation is missing a few required material<br>• Unclear transitions between slides/topics	• Presentation is mostly clear and concise<br>• All required material is presented, though it may have slight organization issues<br>• Transitions between slides/topics are mostly effective	• Presentation is concise and clear<br>• Presentation is easy to follow and well though out<br>• Excellent transitions between slides/topics	
Content and Knowledge	• The majority of the background/project goal is misunderstood or missing<br>• Research plan and experimental approach is mostly misunderstood or missing<br>• Conclusions are misunderstood or missing	• Background/project goal is unclear or missing significant portions<br>• Research plan and experimental approach is missing significant portions or is misunderstood<br>• Analysis of results has a few errors or misconceptions<br>• Conclusions are missing portions of results and are incorrect	• Demonstrating an adequate understanding of background/project goal<br>• Has an adequate research plan and experimental approach<br>• Adequate analysis of results<br>• Conclusions consider the majority of results, though there may be slight confusion	• Demonstrating clear understanding of background and project objective<br>• Well developed research plan and experimental approach,<br>• Excellent critical analysis of results<br>• Well developed conclusions that consider are results	
Delivery	• Speaker has minimal eye contact<br>• Speaker is not engaging<br>• Speaker is hard to understand and has little energy<br>• Confusing presentation flow and poor attitude<br>• Significantly poorly timed	• Speaker has some eye contact with audience<br>• Speaker slightly keeps audience engaged<br>• Speaker has a low energy and volume<br>• Monotone and poor flow<br>• Length is too long/short	• Speaker mostly maintains eye contact with audience<br>• Speaker mostly keeps audience engaged<br>• Speaker has a clear voice for the majority of the presentation<br>• Minimal or too many gestures/inflections/movements<br>• Length is within limits/slightly off limits	• Speaker maintains eye contact with audience throughout the entire presentation<br>• Speaker keeps audience engaged<br>• Speaker has a clear, animated voice<br>• Gestures/inflections/hand movements are used appropriately<br>• Length of presentation is within limits	
Use of Visual Aids	• Several significant issues with slide design<br>• Unclear images that are misplaced<br>• Graphs have illogical organization/flaws	• Slides have a few issues with design and have a few issues in focus/flow<br>• Images are a bit unclear and slightly misplaced<br>• Graphs are slightly confusing and have design flaws (misspellings, blurry, etc.)	• Slides are mostly well designed with a general clear focus/flow<br>• Images are mostly well placed<br>• Graphs are mostly well prepared and mostly demonstrate intended purpose<br>• Figures are mostly explained well	• Slides are well designed with clear focus and flow<br>• Images are well placed<br>• Graphs are well prepared and clearly demonstrate intended purpose<br>• Figures are explained well	
Ability to answer questions	• No to little effort is made to answer questions<br>• Poorly responds to questions	• Several questions are not anticipated and a significant lack of knowledge is observed<br>• Attempts to listen to questions and understands a few<br>• Attempts to provide responses	• All questions are not anticipated and a slight lack of knowledge is observed<br>• Actively listens and understands the majority of questions<br>• Provides thorough responses to most questions with confidence	• Anticipates questions and demonstrates complete knowledge of topic<br>• Actively listens and understands all questions<br>• Provides thorough responses to all questions with confidence	
					/20

Student Exit Questionnaire

Please describe three concepts that you learned during literature review and this research project.

How do you feel about the concepts learned throughout this research project and how they compare to your regular courses?

Do you feel they are similar/different? Do you think they compliment each other?

List three aspects of this project that you think were beneficial. (This could include concepts, lab skills, research approach, etc.)

Is there anything you would change about this research project that would have made your experience better?

What was your overall satisfaction with the research project? Did you enjoy the combination of experiments and simulations?

Do you think coupling experiments and simulations led to improved understanding of results?

Compared to the beginning of this project, what is your understanding of experimental research, computational research, and career as a graduate student/faculty?

How do you think participating in this research project prepared you for your future courses and/or informed you on career aspirations?

Would you recommend this experience to colleagues? Why or why not?"

Representative Experimental and Computational Data

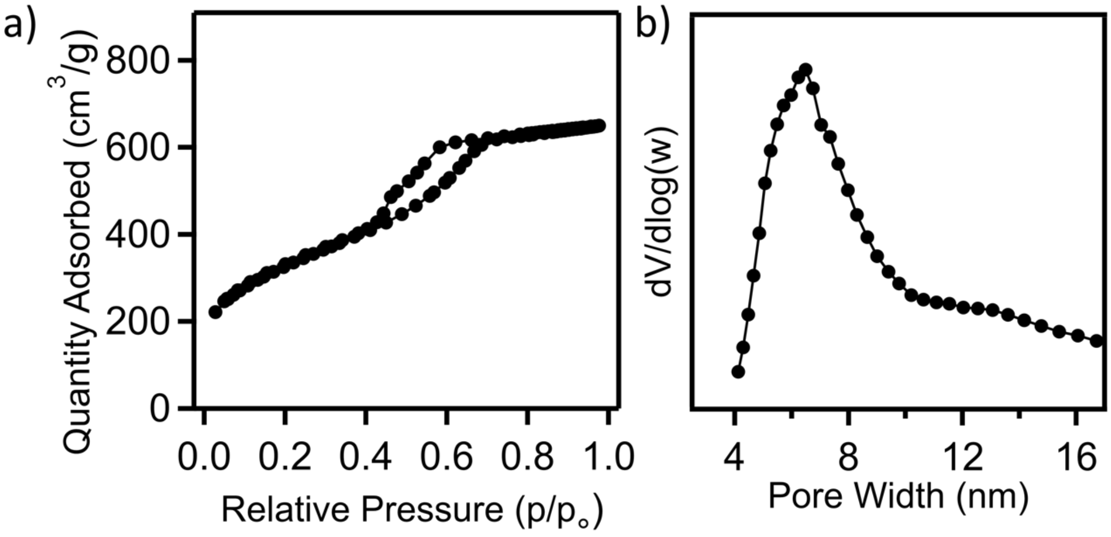

Figure S1. (a) Nitrogen desorption isotherm and (b) corresponding pore size distribution for undoped OMC.

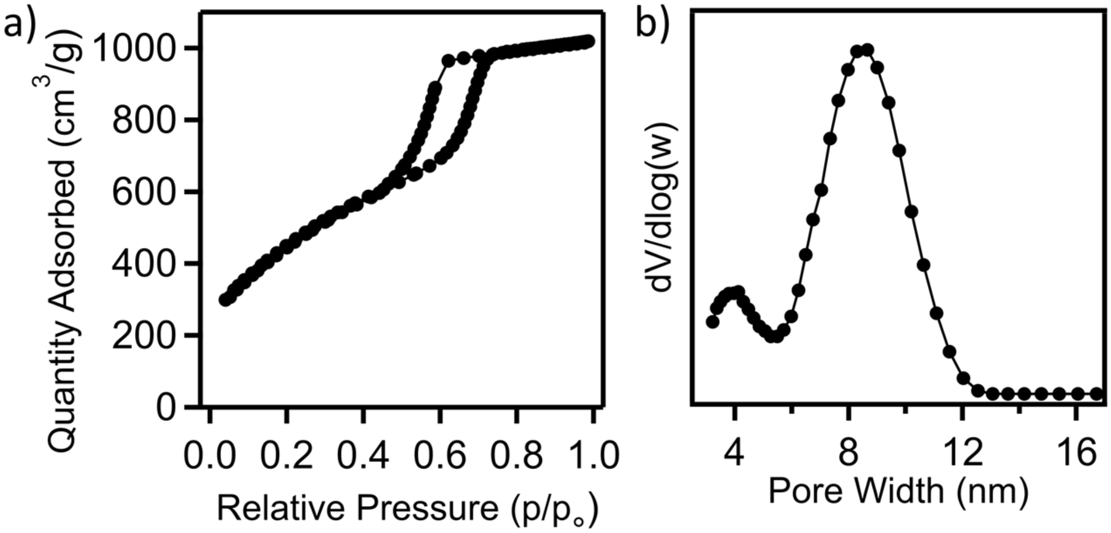

Figure S2. (a) Nitrogen desorption isotherm and (b) corresponding pore size distribution for N-OMC-7.

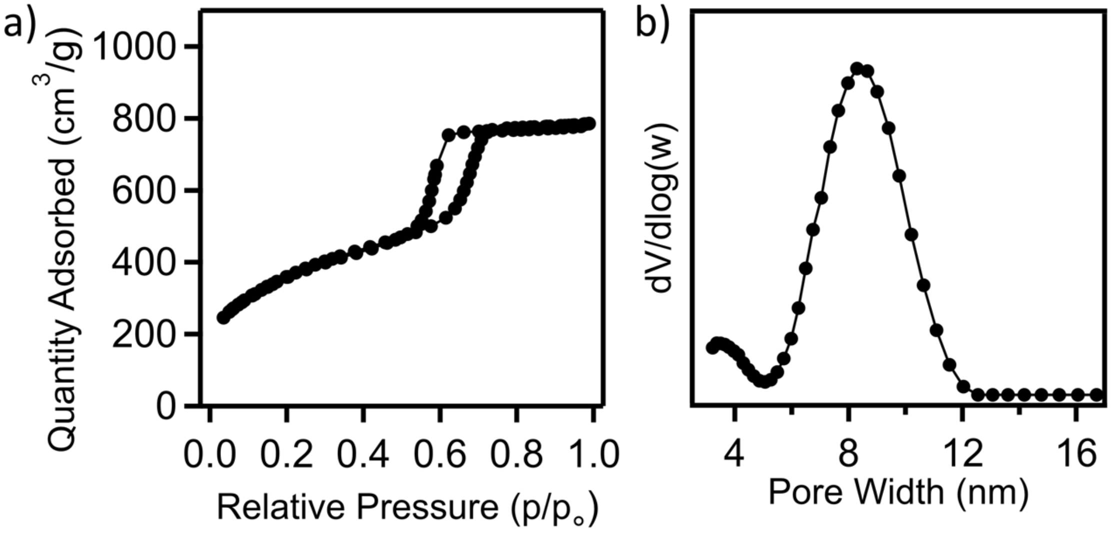

Figure S3. (a) Nitrogen desorption isotherm and (b) corresponding pore size distribution for N-OMC-9.

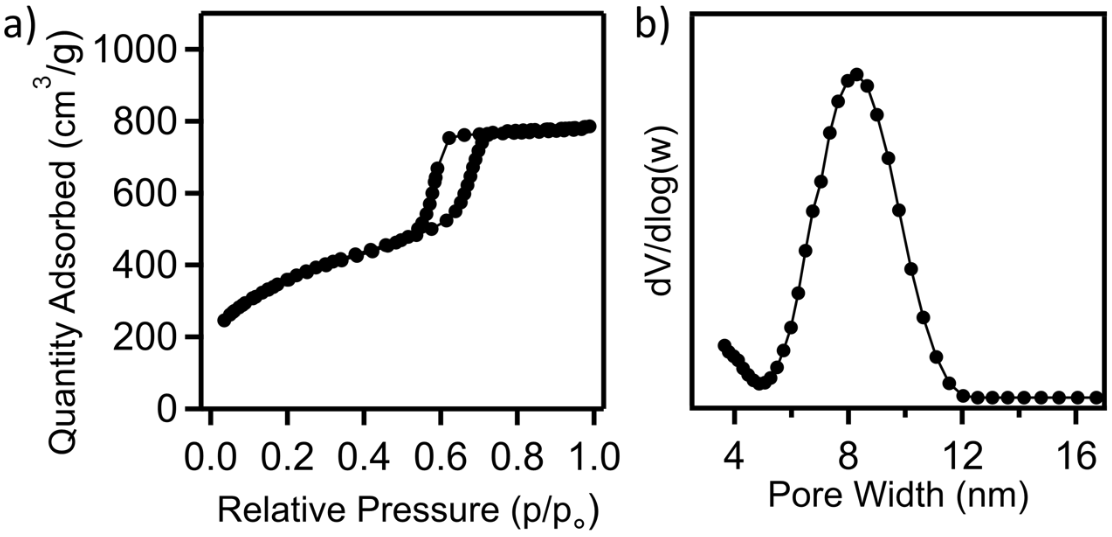

Figure S4. (a) Nitrogen desorption isotherm and (b) corresponding pore size distribution for N-OMC-11.

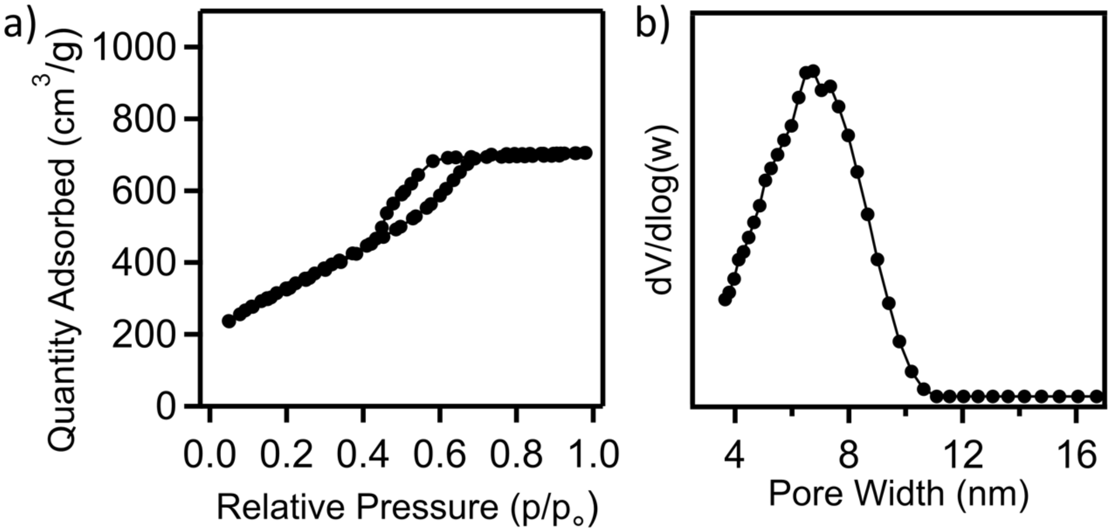

Figure S5. (a) Nitrogen desorption isotherm and (b) corresponding pore size distribution for B-OMC-10.

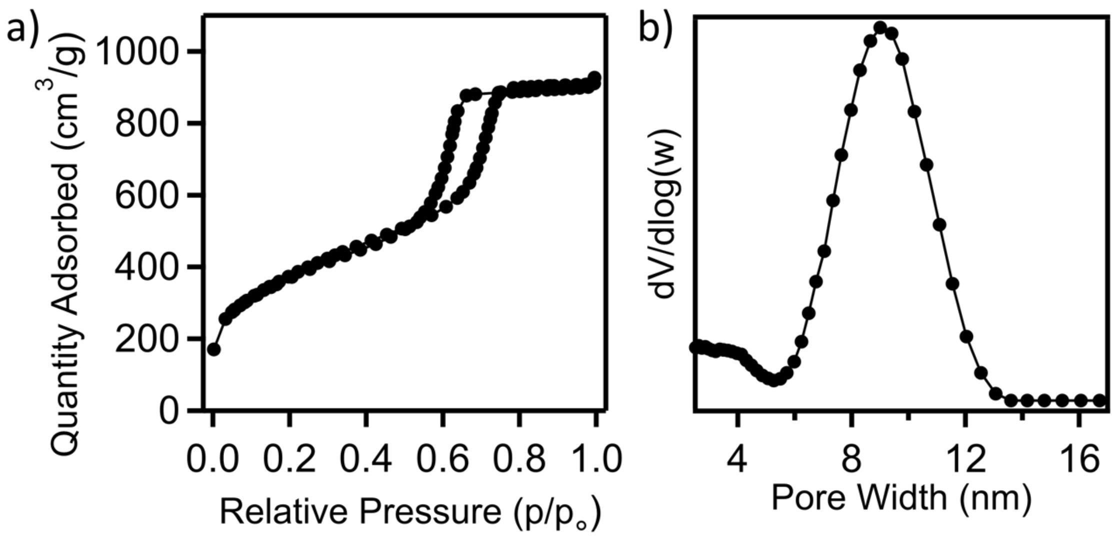

Figure S6. (a) Nitrogen desorption isotherm and (b) corresponding pore size distribution for P-OMC-6.

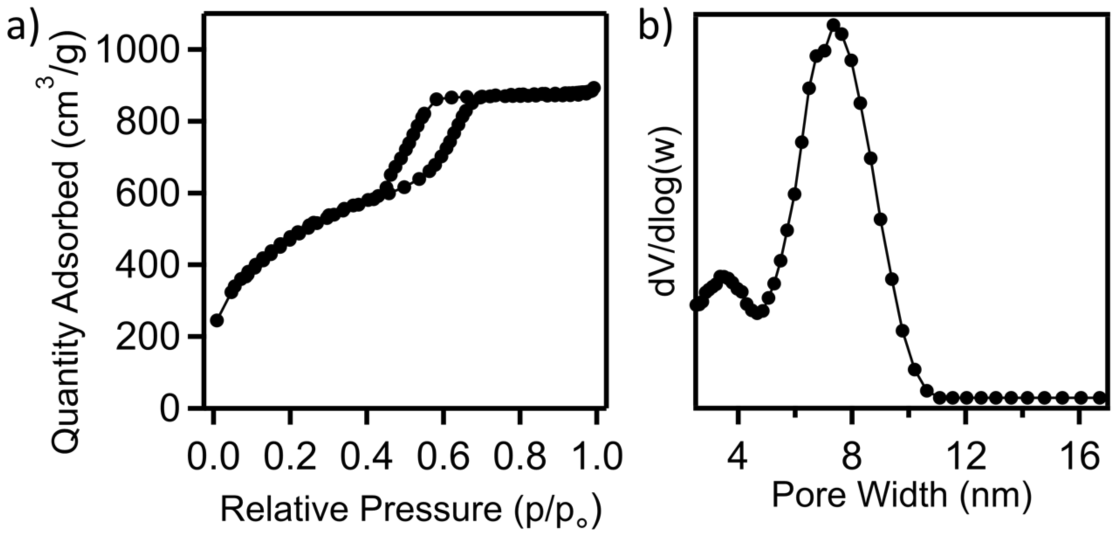

Figure S7. (a) Nitrogen desorption isotherm and (b) corresponding pore size distribution for S-OMC-3.

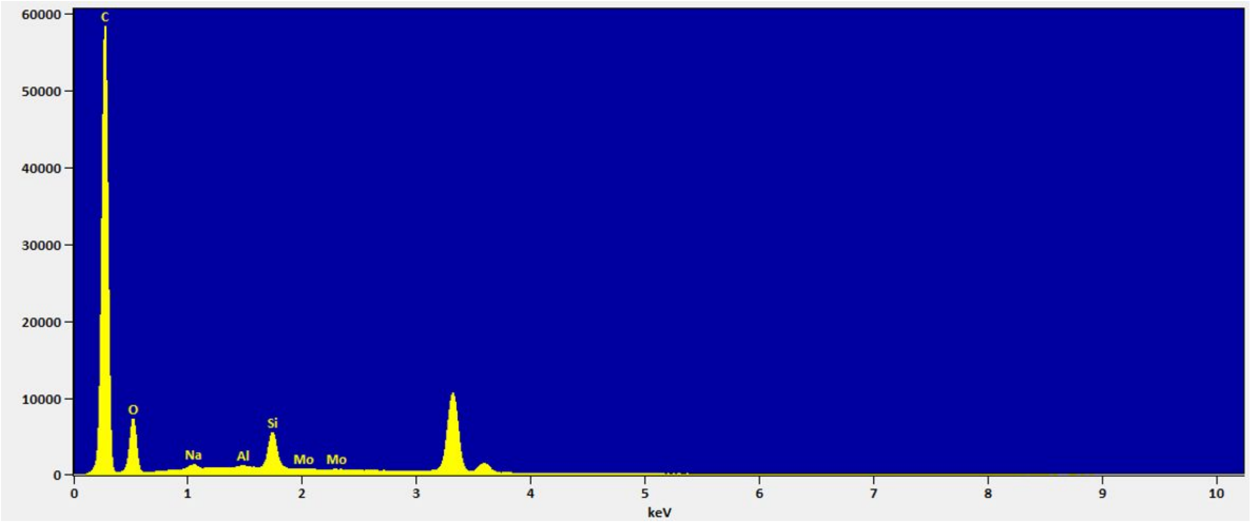

Figure S8. EDX elemental spectra of undoped OMC.

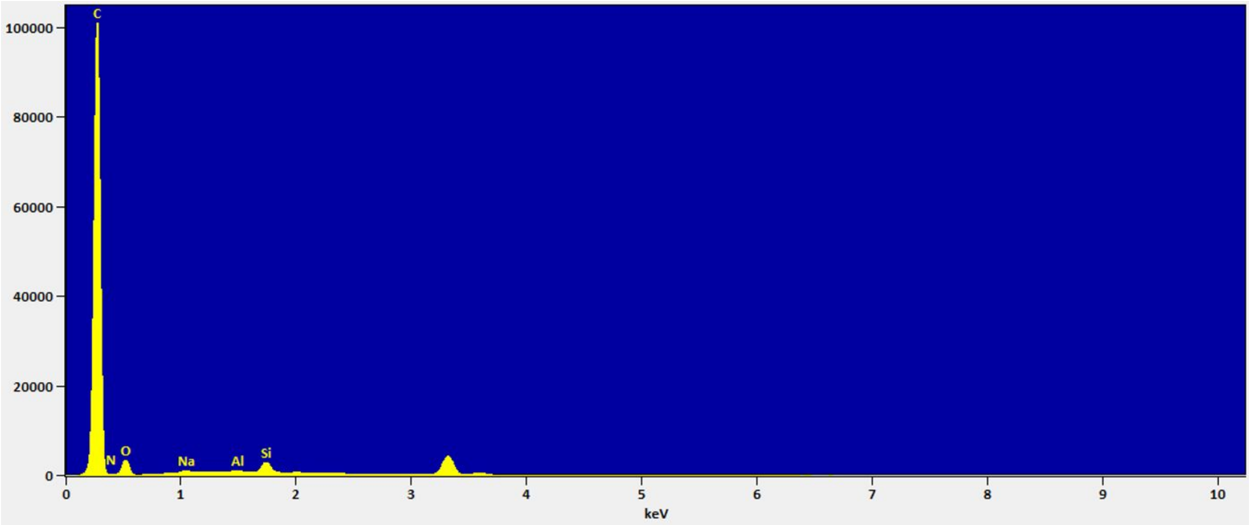

Figure S9. EDX elemental spectra of N-OMC-7.

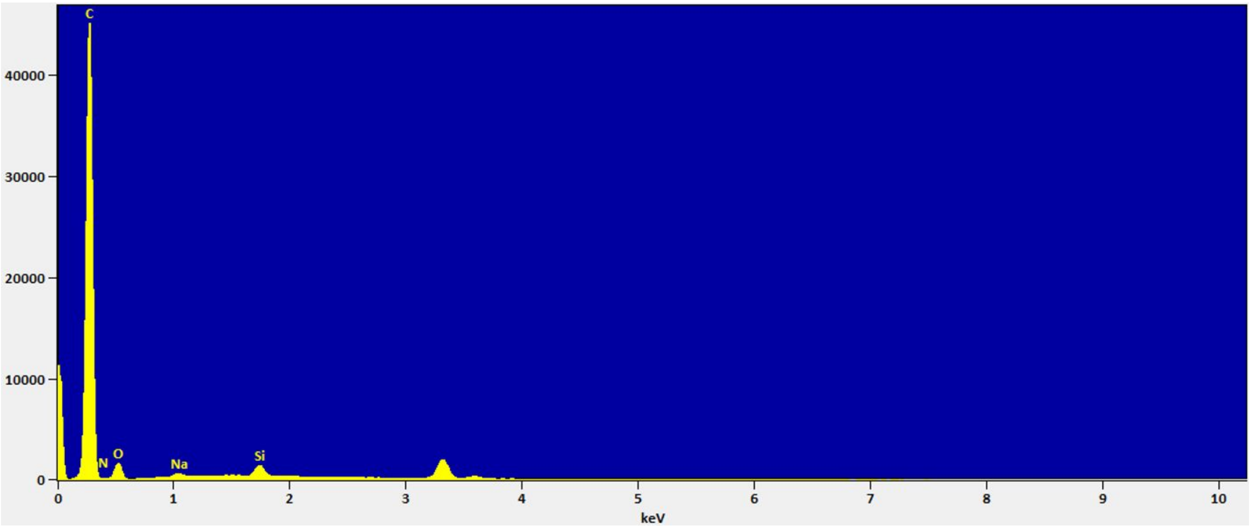

Figure S10. EDX elemental spectra of N-OMC-9.

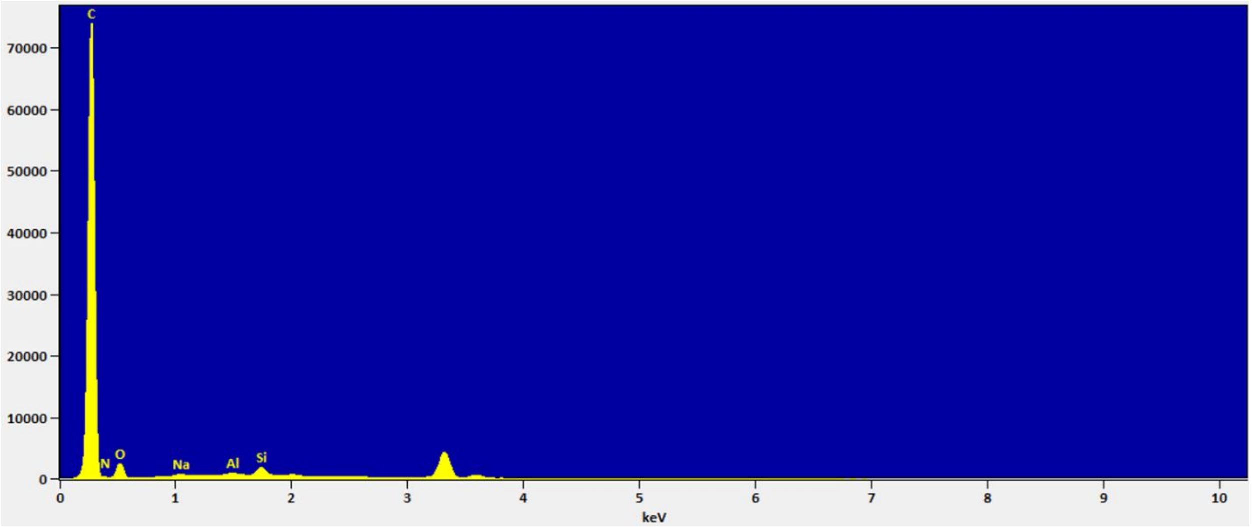

Figure S11. EDX elemental spectra of N-OMC-11.

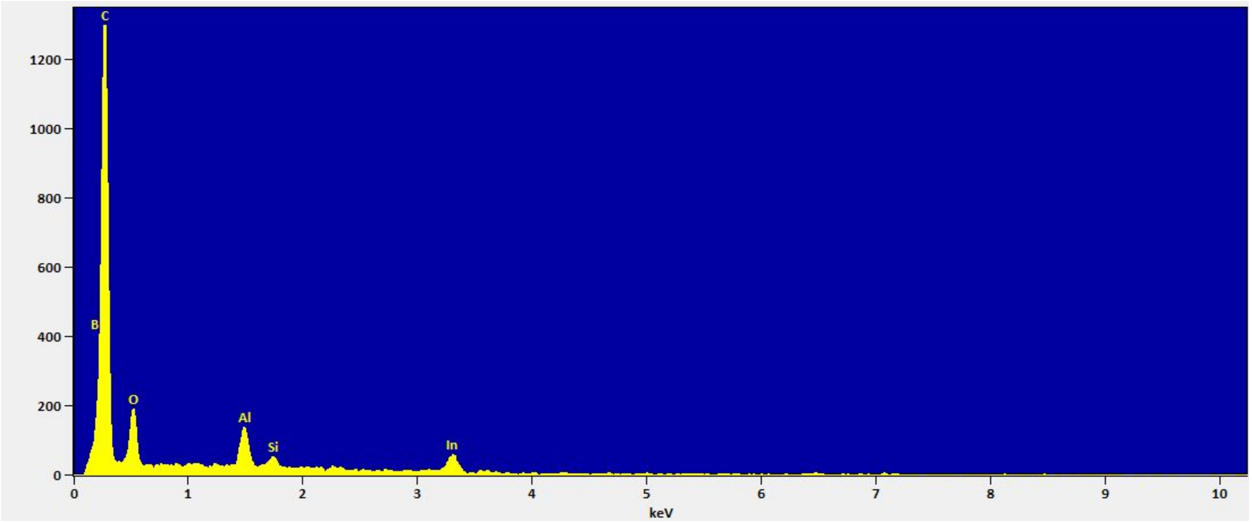

Figure S12. EDX elemental spectra of B-OMC-10.

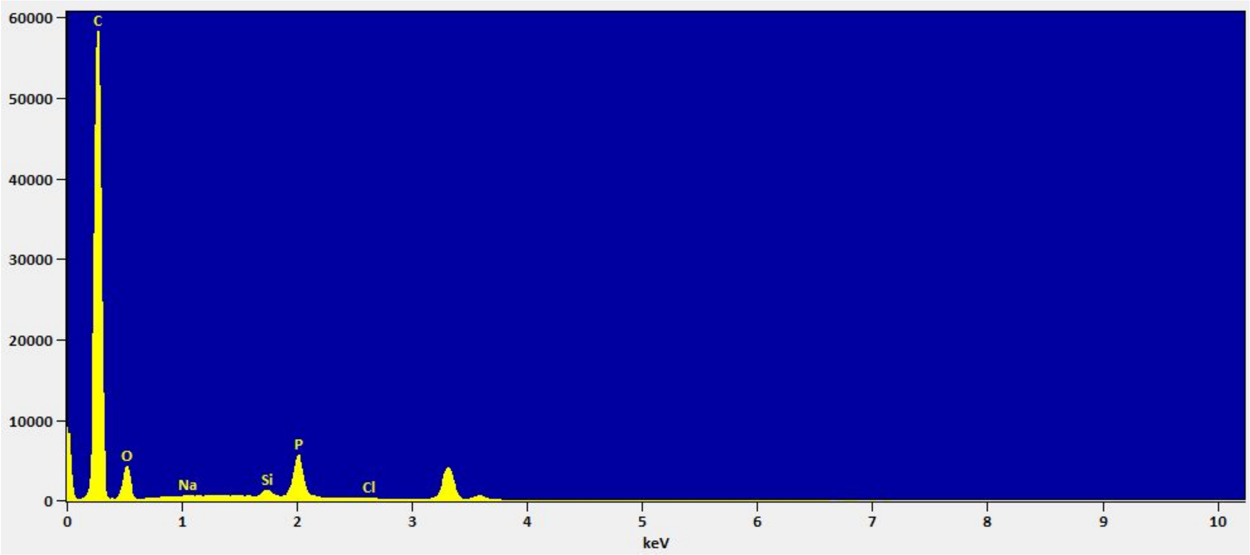

Figure S13. EDX elemental spectra of P-OMC-6.

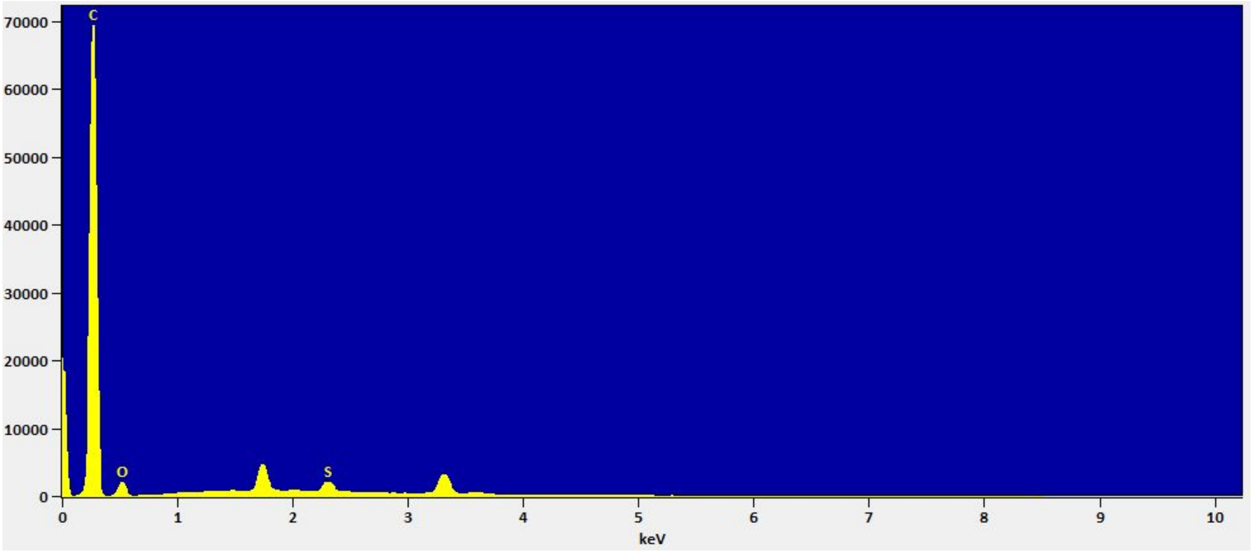

Figure S14. EDX elemental spectra of S-OMC-3.

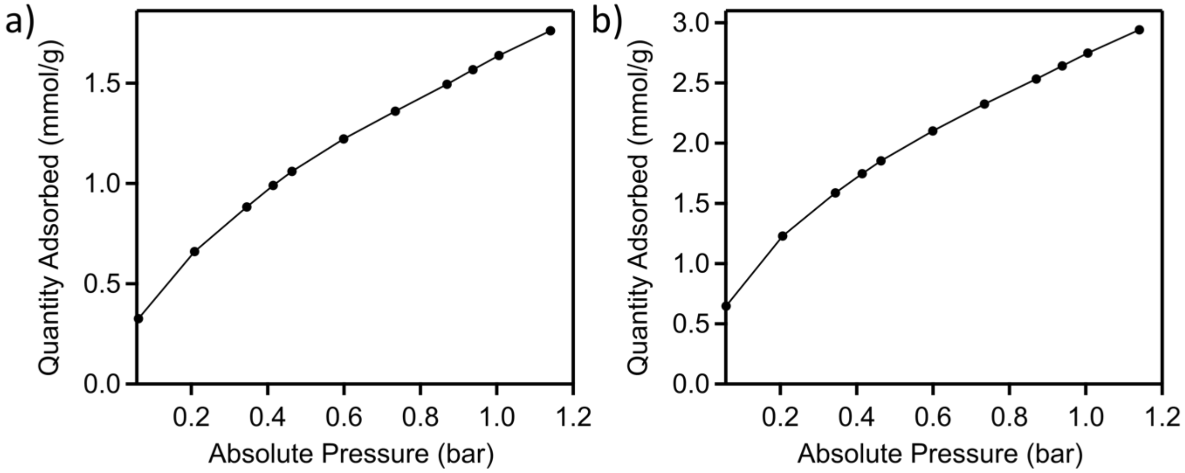

Figure S15. CO_2 adsorption performance at (a) 25 °C and B) 0 °C for undoped OMC.

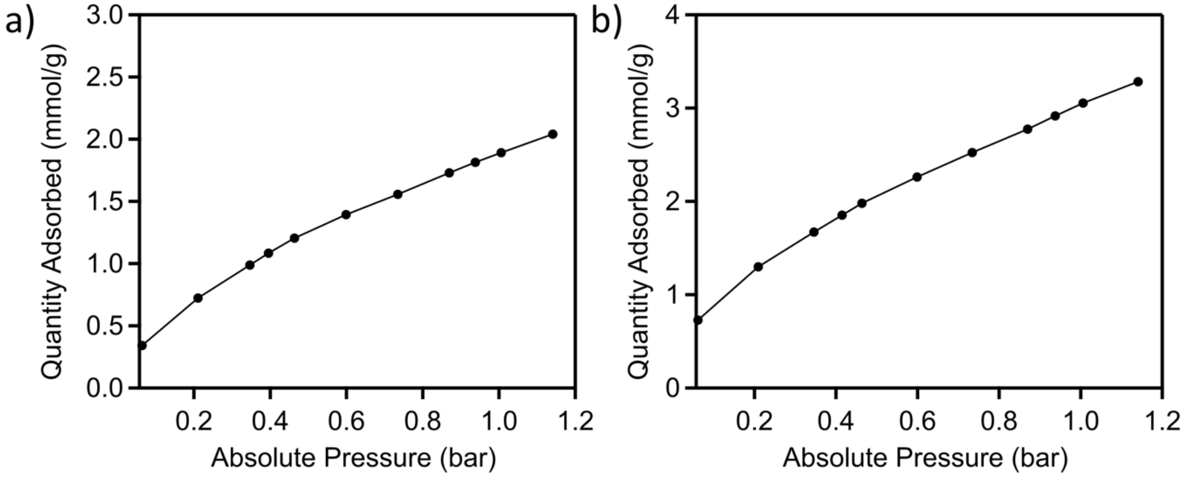

Figure S16. CO_2 adsorption performance at (a) 25 °C and B) 0 °C for N-OMC-7.

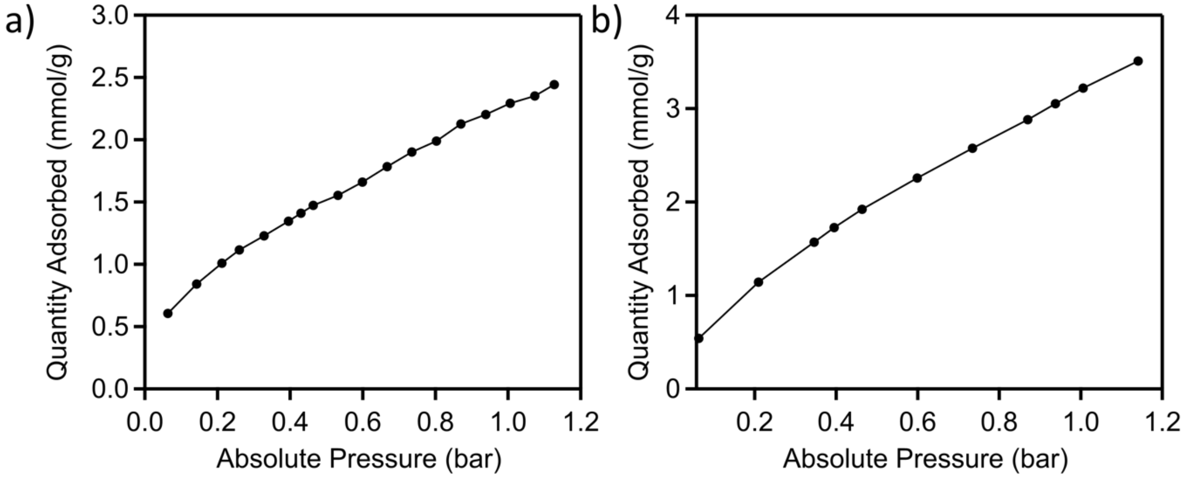

Figure S17. CO_2 adsorption performance at (a) 25 °C and B) 0 °C for N-OMC-9.

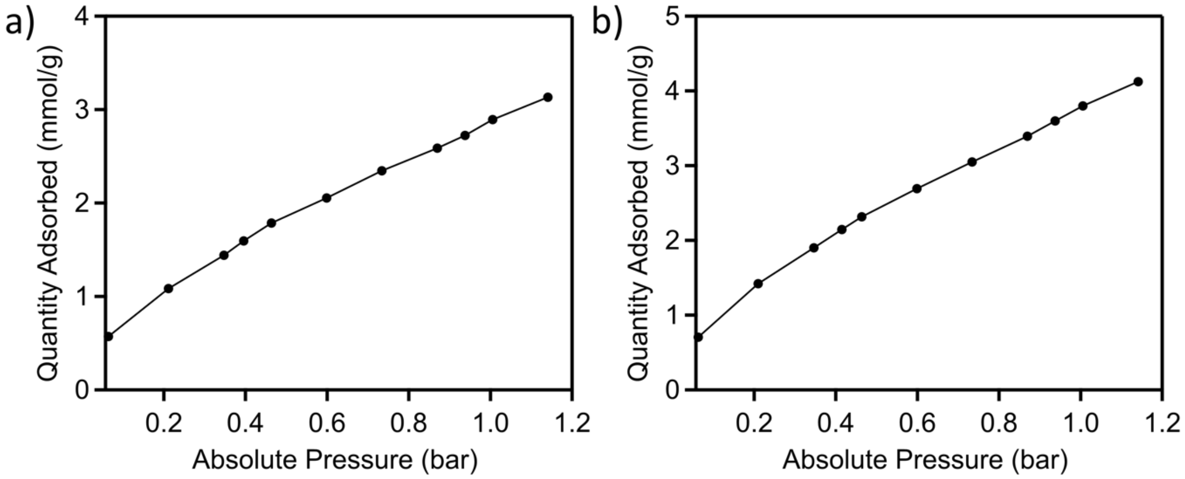

Figure S18. CO_2 adsorption performance at (a) 25 °C and B) 0 °C for N-OMC-11.

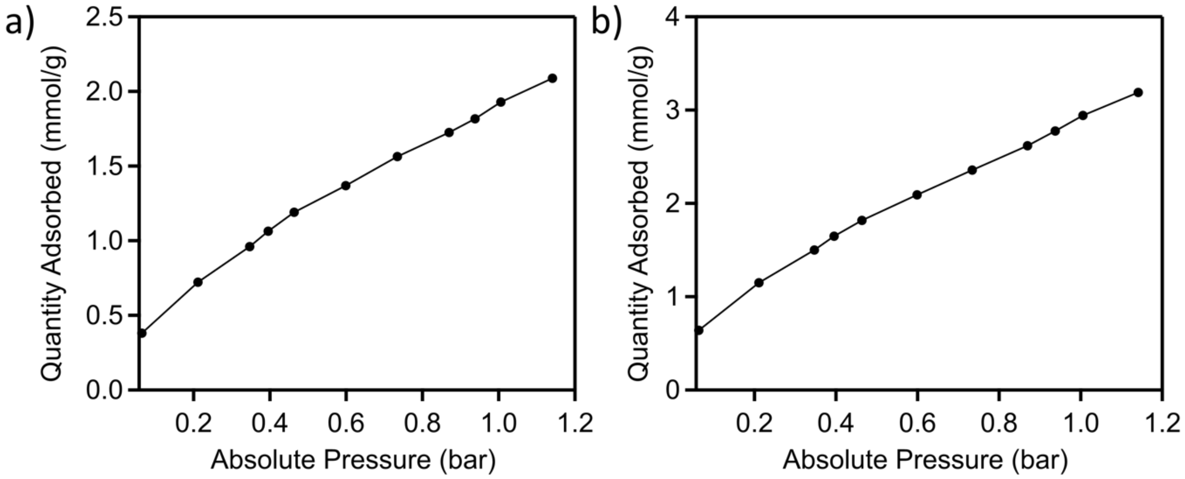

Figure S19. CO_2 adsorption performance at (a) 25 °C and B) 0 °C for B-OMC-10.

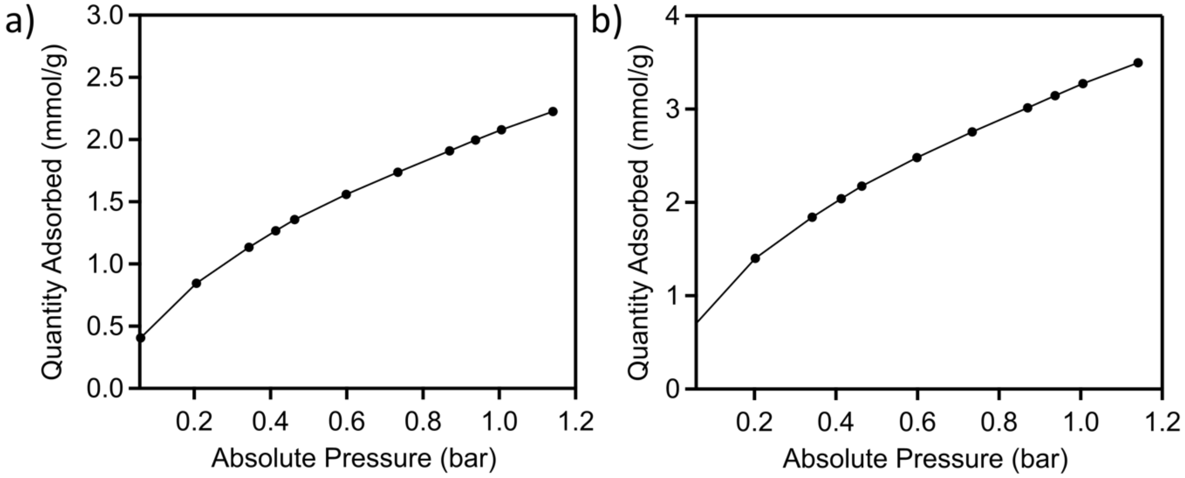

Figure S20. CO_2 adsorption performance at (a) 25 °C and B) 0 °C for P-OMC-6.

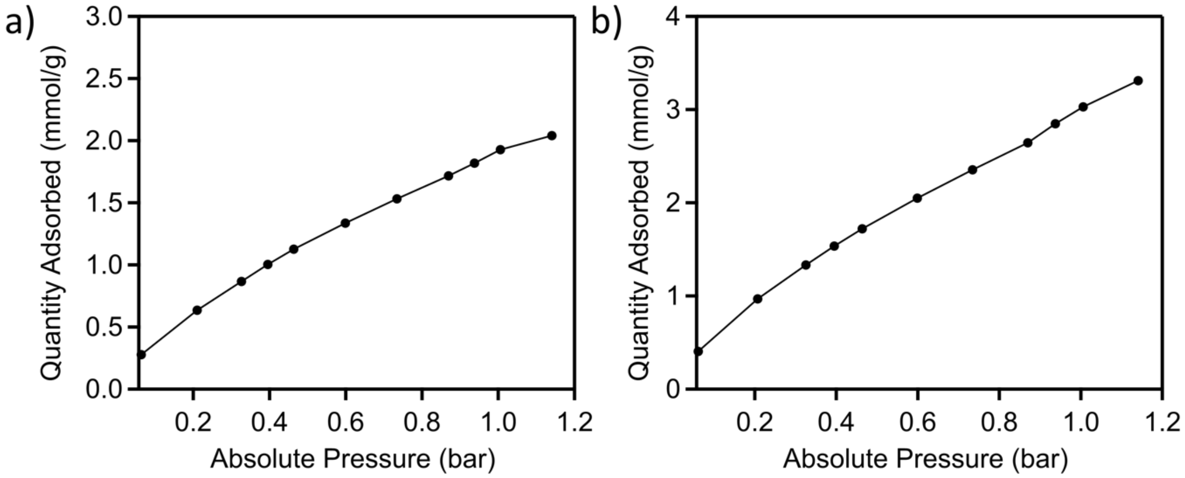

Figure S21. CO_2 adsorption performance at (a) 25 °C and B) 0 °C for S-OMC-3.

Table S1. Cartesian Coordinates of All Structures Shown in Figure 3.

CO2 @ C

C	3.849941	-0.684438	0.013143
C	2.635418	-1.420962	-0.111304
C	3.849965	0.684329	0.013129
C	1.427990	3.531478	-0.222625
C	2.635466	1.420892	-0.111330

C	0.186216	2.842413	-0.352302
C	1.417922	-0.713886	-0.240234
C	0.187702	-1.428356	-0.364273
C	1.417948	0.713853	-0.240252
C	-1.042928	0.714710	-0.493549
C	2.607331	-2.846705	-0.107706
C	-3.478716	-0.684104	-0.716401
C	1.427882	-3.531515	-0.222619
C	0.186132	-2.842410	-0.352311
C	0.187747	1.428359	-0.364287
C	2.607421	2.846636	-0.107734
C	-1.042951	-0.714674	-0.493545
C	-3.478695	0.684220	-0.716378
C	-2.263666	1.421031	-0.601376
C	-2.237145	2.846586	-0.586334
C	-2.237226	-2.846510	-0.586395
C	-1.057351	3.530761	-0.467694
C	-1.057455	-3.530718	-0.467741
C	-2.263710	-1.420952	-0.601412
H	4.787262	-1.235580	0.111286
H	-3.181903	3.387031	-0.670765
H	1.421313	4.623197	-0.215819
H	-4.417499	1.235268	-0.798057
H	4.787303	1.235444	0.111266
H	-4.417532	-1.235127	-0.798102
H	-1.052688	-4.622447	-0.457130
H	1.421172	-4.623233	-0.215824
H	-1.052551	4.622490	-0.457064
H	3.550763	-3.387119	-0.008357

H	-3.181995	-3.386933	-0.670853
H	3.550869	3.387023	-0.008393
C	-1.317855	-0.000011	2.496835
O	-0.165407	-0.000036	2.596256
O	-2.471946	0.000012	2.416993

CO_2 @ N1

C	-3.602998	1.264906	-0.017220
C	-2.291153	1.759376	-0.146523
C	-3.848290	-0.083745	-0.002969
C	-1.941308	-3.275141	-0.203881
C	-2.784427	-0.995988	-0.117128
C	-0.596703	-2.808177	-0.332961
N	-1.229914	0.881659	-0.267072
C	0.079110	1.373689	-0.390305
C	-1.457468	-0.500490	-0.249933
C	0.954442	-0.933273	-0.484194
C	-2.048856	3.160968	-0.157002
C	3.572583	0.044065	-0.670542
C	-0.789260	3.656172	-0.275208
C	0.314110	2.767957	-0.391528
C	-0.378411	-1.400954	-0.360288
C	-2.989652	-2.408254	-0.101447
C	1.171840	0.470952	-0.500952
C	3.337864	-1.305510	-0.652437
C	2.022343	-1.864072	-0.558889
C	1.860014	-3.300664	-0.542508
C	2.703305	2.379469	-0.595477
C	0.514824	-3.680370	-0.431024
C	1.646752	3.245309	-0.500904

C	2.497258	0.975495	-0.593104
H	-4.405832	1.994687	0.072202
H	3.723615	2.759142	-0.670923
H	4.155179	-2.026836	-0.705885
H	-4.866137	-0.463647	0.098629
H	-0.606889	4.731817	-0.279522
H	-2.111250	-4.353627	-0.187218
H	1.806560	4.324401	-0.500923
H	4.589823	0.434116	-0.737558
H	0.256411	-4.749264	-0.410417
H	-2.915893	3.813034	-0.063076
H	-4.013508	-2.772139	-0.001558
C	1.152221	-0.056634	2.491758
O	0.018668	0.187916	2.461887
O	2.282393	-0.284643	2.533583

CO_2 @ N2

C	-0.955146	-0.821048	-0.613774
C	-1.129261	0.555663	-0.614670
C	0.285326	-1.415681	-0.453742
C	-0.076019	1.440677	-0.455063
C	1.406076	-0.545597	-0.308708
C	1.226124	0.877309	-0.309411
C	-2.197736	-1.436405	-0.663089
C	-2.485970	0.842135	-0.664253
C	0.331595	-2.816740	-0.375989
C	-0.380318	2.809128	-0.378158
C	2.672566	-1.116800	-0.105138
C	2.310337	1.746127	-0.106278
C	-2.168082	-2.854060	-0.592430

N	-3.140240	-0.398169	-0.721997
C	0.753807	3.672854	-0.188631
C	3.769919	-0.219074	0.070837
C	1.645089	-3.370576	-0.185939
C	2.025110	3.159213	-0.066722
C	2.748360	-2.556369	-0.064547
C	-0.929467	-3.494969	-0.462425
C	3.596686	1.150137	0.070385
C	-2.810422	2.222473	-0.594287
C	-1.770523	3.151771	-0.464697
H	-3.075624	-3.458048	-0.623504
H	2.861235	3.845620	0.083695
H	4.769271	-0.632517	0.223236
H	-0.929994	-4.585585	-0.404472
H	3.729138	-3.012718	0.086157
H	0.605380	4.753311	-0.131085
H	-3.839839	2.581244	-0.625426
H	1.770470	-4.453917	-0.127703
H	4.461484	1.799616	0.222579
H	-2.042661	4.207933	-0.407289
H	-4.141948	-0.524867	-0.696749
C	-1.334288	-0.164066	2.382341
O	-0.194259	-0.021680	2.507744
O	-2.479219	-0.307007	2.282364

CO_2 @ N3

C	2.391261	3.379841	-0.000006
C	2.797548	2.011503	-0.000031
C	1.066399	3.729801	0.000048
C	-2.313049	2.079702	0.000167

C	0.041202	2.737468	0.000084
C	-1.945928	0.700951	0.000137
C	1.801824	1.009600	0.000007
C	2.174186	-0.369809	-0.000019
C	0.423109	1.378384	0.000066
C	-0.205479	-1.006053	0.000077
C	4.168332	1.614671	-0.000094
C	0.472225	-3.725863	0.000013
C	4.525048	0.292007	-0.000119
C	3.537820	-0.738049	-0.000084
C	-0.576422	0.364917	0.000099
C	-1.351050	3.056505	0.000138
C	1.167999	-1.380960	0.000014
C	-0.848855	-3.359376	0.000075
C	-1.217721	-1.983064	0.000114
C	-2.570302	-1.519513	0.000186
C	2.900542	-3.094752	-0.000085
N	-2.916396	-0.260843	0.000184
C	3.870119	-2.126023	-0.000114
C	1.516205	-2.749602	-0.000019
H	3.162494	4.152579	-0.000032
H	-3.386413	-2.252150	0.000214
H	-3.376784	2.319315	0.000193
H	3.178535	-4.150388	-0.000113
H	0.776518	4.782354	0.000069
H	-1.634232	-4.117737	0.000109
H	4.924862	-2.408397	-0.000163
H	-1.642942	4.108635	0.000146
H	5.578892	0.006925	-0.000169

H	4.936522	2.390427	-0.000123
H	0.748327	-4.782034	-0.000012
C	-5.596254	-0.002880	-0.000254
O	-5.687309	-1.156790	0.000074
O	-5.590636	1.154282	-0.000548

CO2 @ B1

C	1.868405	3.194065	-0.540693
C	2.170810	1.838630	-0.584074
C	0.530028	3.689707	-0.423988
C	-2.995232	2.533518	-0.099080
C	-0.593122	2.875040	-0.333491
C	-2.825640	1.084534	-0.117836
B	0.988141	0.900324	-0.504147
C	1.199313	-0.589458	-0.514244
C	-0.414597	1.443144	-0.366177
C	-1.248704	-0.895327	-0.278199
C	3.475433	1.220323	-0.676851
C	-0.857027	-3.698288	-0.279224
C	3.621136	-0.141247	-0.686665
C	2.509912	-1.081971	-0.602987
C	-1.470214	0.555433	-0.258496
C	-1.944944	3.382548	-0.199630
C	0.074875	-1.445890	-0.402310
C	-2.121276	-3.168727	-0.165817
C	-2.330821	-1.766134	-0.159737
C	-3.729294	-1.242914	-0.018405
C	1.610697	-3.340965	-0.499988
C	-3.876973	0.233418	-0.008435
C	2.693261	-2.496821	-0.596792

C	0.273625	-2.847491	-0.396253
H	2.666860	3.944799	-0.591204
H	1.763981	-4.422679	-0.495008
H	-2.106814	4.462618	-0.178492
H	0.388234	4.772654	-0.397075
H	4.625787	-0.565930	-0.750934
H	-4.012010	2.918345	0.003490
H	3.701161	-2.912026	-0.666897
H	-0.712265	-4.781029	-0.276954
H	-4.887579	0.638850	0.095587
H	4.375554	1.841604	-0.734616
H	-2.985254	-3.830423	-0.073329
H	-4.358547	-1.661720	-0.826609
H	-4.176820	-1.652580	0.907007
C	1.229860	0.033248	2.522988
O	2.360353	0.269323	2.468278
O	0.099640	-0.202368	2.609730

CO_2 @ B2

C	-3.866725	1.097775	0.247893
C	-3.128391	-0.124097	0.127764
C	-3.252383	2.312005	0.150895
C	0.187973	3.713365	-0.387580
C	-1.840700	2.410526	-0.077294
C	0.959084	2.535255	-0.522933
C	-1.736964	-0.058189	-0.101475
C	-0.975164	-1.259880	-0.219697
C	-1.090924	1.214659	-0.206345
C	1.072325	0.071527	-0.567556
C	-3.748279	-1.398973	0.234714

C	2.587239	-2.289189	-0.776286
C	-3.015898	-2.554022	0.123311
C	-1.612778	-2.514864	-0.104436
C	0.310067	1.293210	-0.435784
C	-1.173143	3.656977	-0.174075
C	0.435633	-1.194690	-0.452190
C	3.208703	-1.063331	-0.891130
C	2.453724	0.119936	-0.787798
O	3.105153	1.297655	-0.888597
C	0.519672	-3.645466	-0.433487
B	2.481387	2.514544	-0.752619
C	-0.825370	-3.706762	-0.218575
C	1.192970	-2.386811	-0.557505
H	-4.942853	1.038144	0.423098
H	-1.757438	4.573609	-0.072468
H	0.688249	4.681871	-0.453908
H	4.281951	-0.974194	-1.059355
H	-3.830125	3.233104	0.247663
H	3.174819	-3.205943	-0.852220
H	-1.326702	-4.672043	-0.126621
H	3.181200	3.486089	-0.842310
H	1.108994	-4.560797	-0.514638
H	-4.824796	-1.445415	0.410603
H	-3.505184	-3.526040	0.210037
C	2.295191	0.046306	2.252100
O	2.501601	1.177416	2.115347
O	2.094469	-1.081658	2.405504

CO_2 @ B3

C	1.045583	3.408377	-0.496500

C	-0.178473	2.788758	-0.395273
O	2.214940	2.770886	-0.592003
C	3.406826	-0.663999	-0.643912
B	2.254634	1.385793	-0.597155
C	2.268231	-1.426607	-0.556441
C	-0.229584	1.346039	-0.395311
C	-1.450975	0.644487	-0.266578
C	0.964731	0.639179	-0.509227
C	-0.207753	-1.479551	-0.351113
C	-1.437519	3.497909	-0.276906
C	-2.700864	-2.971943	-0.073067
C	-2.608060	2.817011	-0.160830
C	-2.669081	1.365826	-0.145259
C	0.985141	-0.766143	-0.483038
O	3.437719	0.674087	-0.673560
C	-1.436938	-0.775771	-0.247613
C	-1.417230	-3.586880	-0.172305
C	-0.201697	-2.913186	-0.305088
C	1.095718	-3.565441	-0.397980
C	-3.832424	-0.763873	0.000674
C	2.262414	-2.877558	-0.513389
C	-3.857886	0.631739	-0.014123
C	-2.641629	-1.519276	-0.110072
H	1.148741	4.495696	-0.501631
H	1.111403	-4.656822	-0.366078
H	4.401137	-1.112912	-0.694789
H	-1.357333	-4.683093	-0.144512
H	-4.756883	-1.335923	0.105084
H	-1.432693	4.589521	-0.279755

H	-4.806776	1.163325	0.079507
H	-3.547020	3.366359	-0.070565
H	3.213789	-3.410063	-0.572997
C	1.206594	0.183463	2.482895
O	2.340868	0.343147	2.302808
O	0.076685	0.034329	2.664536

CO_2 @ P1

C	2.501677	1.102428	-1.028263
C	1.107804	1.275310	-1.002378
P	0.146378	-0.132724	-1.034315
C	0.912705	-1.607260	-0.738435
C	-1.397494	-0.022823	-0.334960
C	-2.019544	-1.214691	0.105792
C	-1.290865	-2.478522	0.048346
C	0.022747	-2.665077	-0.307174
C	-1.898132	1.294995	-0.179925
H	0.437657	-3.672596	-0.217962
C	2.316087	-1.494305	-0.882853
C	2.982522	-0.256781	-1.016079
C	-3.304725	-1.057052	0.649421
H	-1.846558	-3.358718	0.378591
C	-3.185663	1.380129	0.369508
C	-3.866319	0.218389	0.753505
C	-1.053676	2.453254	-0.491521
H	-3.856436	-1.927796	1.008309

H	-3.649390	2.356722	0.519983
H	-4.865740	0.316220	1.180195
H	0.789176	3.420985	-0.937635
C	0.274956	2.460465	-0.839302
H	2.923162	-2.401255	-0.791831
H	4.073889	-0.365803	-1.018883
H	-1.538489	3.423504	-0.362417
O	1.254997	0.207801	1.933826
O	3.536286	0.503729	2.178514
C	2.399055	0.362767	2.037259

CO2 @ P2

C	3.166129	1.324648	-0.270513
C	1.789850	1.434504	-0.136200
P	0.908942	0.025965	0.546315
H	1.421258	3.542338	-0.603506
C	1.834304	-1.414279	0.004088
C	-0.517091	-0.048341	-0.518959
C	-1.055891	-1.307899	-0.830218
C	-0.289533	-2.537147	-0.576853
C	1.035537	-2.593625	-0.276357
C	-1.094326	1.157217	-0.951117
H	1.532333	-3.568152	-0.254819
C	3.206695	-1.274775	-0.142326
C	3.573514	0.016693	-0.496383

C	-2.295983	-1.340707	-1.484849
H	-0.821733	-3.476381	-0.743849
C	-2.334288	1.086926	-1.603528
C	-2.932838	-0.151094	-1.840574
C	-0.367176	2.428778	-0.820403
H	-2.753508	-2.301323	-1.729136
H	-2.821277	2.004281	-1.939855
H	-3.900694	-0.190904	-2.342189
H	3.802725	2.184253	-0.509315
C	0.955061	2.555377	-0.529278
H	3.870723	-2.133105	-0.295775
H	-0.928481	3.330139	-1.076812
O	0.610669	0.093492	2.008891
C	-2.061721	0.156513	2.187979
O	-2.076139	1.312270	2.129749
O	-2.123146	-0.997932	2.238043

CO2 @ P3

C	2.681720	1.079693	-0.548161
C	3.444612	-0.110729	-0.481062
C	1.301817	0.995472	-0.593613
C	0.683097	-2.713665	-0.521544
C	-1.442430	-1.483838	-0.534629
C	0.714249	-0.286076	-0.582655
C	-2.851114	-1.364347	-0.481491

C	-3.444593	-0.111055	-0.480930
C	2.851228	-1.364067	-0.481446
C	-0.682850	-2.713742	-0.521623
H	3.188796	2.046657	-0.561079
C	1.442552	-1.483677	-0.534465
C	-2.681821	1.079450	-0.548086
H	4.532311	-0.037588	-0.433560
H	3.469670	-2.262842	-0.437065
C	-1.301930	0.995323	-0.593804
C	-0.714252	-0.286163	-0.582813
P	-0.000080	2.312845	-0.787008
H	-3.469490	-2.263164	-0.437085
H	-1.225172	-3.661106	-0.496858
H	1.225516	-3.660974	-0.496756
H	-3.188971	2.046373	-0.560931
H	-4.532294	-0.038034	-0.433264
O	-0.000283	3.106611	0.657813
H	0.001013	2.504932	1.416774
C	0.000074	-0.349071	2.439037
O	-0.000335	0.811111	2.447536
O	0.000326	-1.501217	2.460733

CO2 @ P4

C	0.935116	-2.724207	-0.352690
C	-0.269377	-3.452176	-0.505886

C	0.885527	-1.344722	-0.365078
C	-2.765172	-0.617581	-0.879271
C	-1.497726	1.476222	-0.700365
C	-0.358725	-0.710290	-0.540211
C	-1.354460	2.884667	-0.676874
C	-0.105198	3.460328	-0.508435
C	-1.489769	-2.818012	-0.675856
C	-2.732846	0.747183	-0.879031
H	1.881703	-3.253890	-0.232102
C	-1.566029	-1.404370	-0.700318
C	1.063478	2.676131	-0.355339
H	-0.232567	-4.542421	-0.494602
H	-2.400367	-3.408451	-0.796074
C	0.948406	1.300538	-0.366722
C	-0.324729	0.725721	-0.540564
P	2.184780	-0.052160	-0.228903
H	-2.236026	3.517622	-0.797117
H	-3.657238	1.312806	-1.011031
H	-3.715324	-1.138783	-1.011254
H	2.034321	3.160311	-0.236244
H	-0.016709	4.547619	-0.498175
O	3.312289	-0.079563	-1.180004
O	2.684679	-0.063277	1.303809
H	1.958972	-0.046239	1.945748

C	-1.027858	0.027701	2.438281
O	0.115575	0.004047	2.635754
O	-2.166582	0.047671	2.267258
